# Nitrification Mechanisms for the P460 Enzymes

**DOI:** 10.1021/acs.jpcb.4c06537

**Published:** 2024-12-18

**Authors:** Per E. M. Siegbahn

**Affiliations:** Department of Organic Chemistry, Arrhenius Laboratory, Stockholm University, Stockholm SE-106 91, Sweden

## Abstract

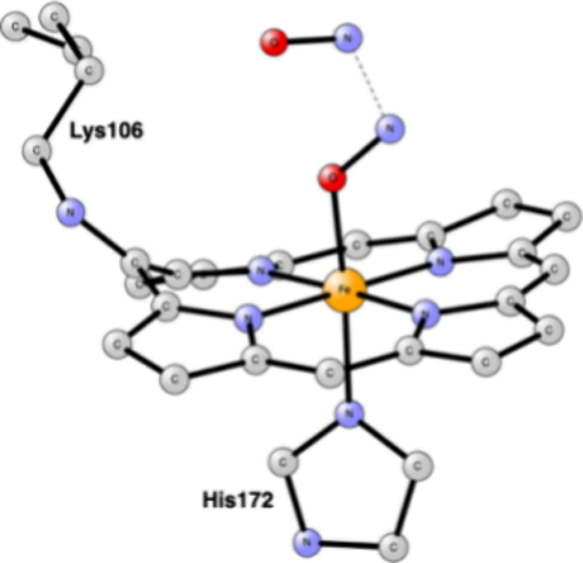

The oxidation of hydroxylamine was studied by quantum chemical modeling. Hydroxylamine is the product of ammonia oxidation in ammonia monooxygenase. That mechanism has been studied recently by quantum chemical modeling as here. Only two enzymes can oxidize hydroxylamine, hydroxylamine oxidase and cytochrome-P460. Both employ the unusual P460-heme cofactor. In hydroxylamine oxidase, there is a covalently linked tyrosine, while in cytochrome-P460, there is a covalently linked lysine. The calculations give explanations for the experimental findings that NO is the final product in hydroxylamine oxidase, while N_2_O is the final product in cytochrome-P460. The effect of the covalent attachments has been investigated, and reasons for their presence have been given. The methodology used, which was proven to give very good agreement with experiments for several redox enzymes, again leads to excellent agreement with experimental findings.

## I Introduction

Nitrification is the process where ammonia is broken down in nature. There are many studies that are indirectly related to the present study on the breakdown of hydroxylamine (NH_2_OH). The formation of ammonia from nitrogen in air is performed by nitrogenase. The mechanism of that process has been studied in detail by experiments over the past 30 years.^1,2^ A mechanism for the activation of the triply bonded N_2_ has been suggested based on spectroscopic information.^2^ A different mechanism has been suggested from model calculations.^3^ The first step in breaking down ammonia is performed by ammonia monooxygenase (AMO). A structure, which is part of particulate methane monooxygenase (pMMO), has recently been obtained.^4^ Based on the experimentally suggested active site, a mechanism has been suggested from model calculations.^5^ The product of ammonia oxidation is hydroxylamine. In this study, the next step, in which the hydroxylamine is oxidized, is modeled by quantum chemical calculations.

**Figure 1:**
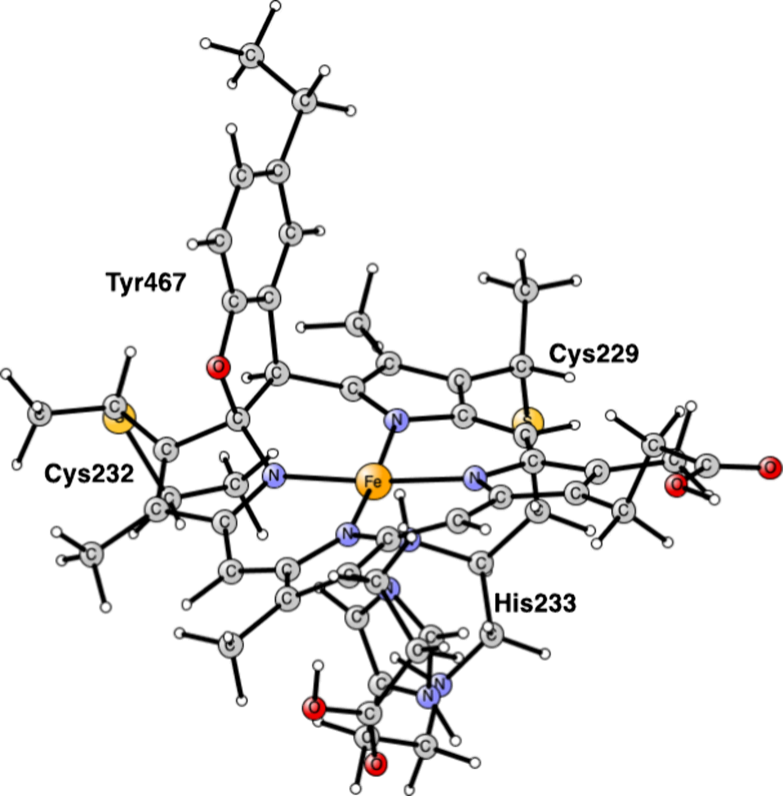
Heme P460 active site for hydroxylamine oxidase (HAO), based on the PDB 4FAS X-ray structure.^9^

**Figure 2:**
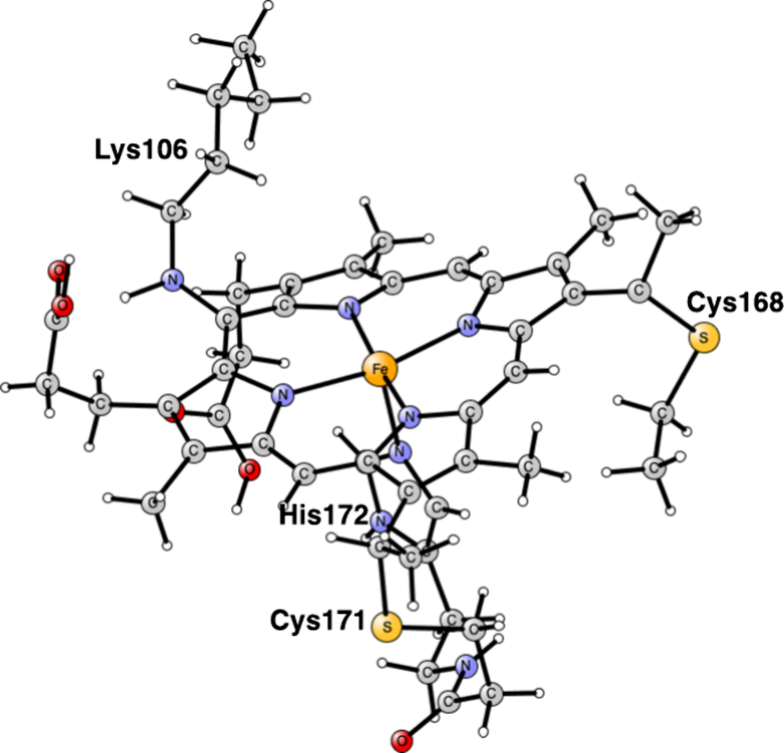
Heme P460 active site for cyt-P460, based on the PDB 6AMG X-ray structure.^10^

Two enzymes can oxidize hydroxylamine, hydroxylamine oxidase (HAO) and cytochrome P460 (cyt-P460).^6^ Interestingly, both of them use the cofactor heme P460, which is used in only these enzymes. However, there are important differences among the cofactors. In HAO there is a covalent cross-link to a tyrosine, see Figure 1, while in Cyt-P460, there is a covalent cross-link to a lysine, see Figure 2.

Both HAO and Cyt-P460 have been studied in detail experimentally.^6−8^ Most importantly, it has been shown that under anaerobic conditions, N_2_O (nitrous oxide) is the exclusive product of NH_2_OH oxidation in cyt-P460, but for HAO, the final product is NO, establishing it as an intermediate of nitrification. Furthermore, it has been shown that the heme–Lys cross-link in cyt-P460 is essential for the enzyme to avoid a catalytic dead end during catalysis. It was suggested that one reason could be to avoid axial histidine dissociation, a common occurrence in heme proteins. It was also shown that a basic residue is absolutely necessary for the oxidation of NH_2_OH by the P460 cofactors.

## II Methods

The methods used here are the same as were used in many similar redox enzymes.^3,5,11,12^ DFT has been used based on the B3LYP functional.^13^ with the fraction of exact exchange reduced to 15%. The geometries were optimized by using a medium-size basis set, lacvp*. For the final energies, a large basis set was used (cc-pvtz(-f)) for the nonmetal atoms, while a lacv3p+ basis was used for iron. For zero-point effects and determination of transition states, Hessians were computed using B3LYP with lacvp*. A dielectric constant of 4.0 was used for the solvation effects,^14^ and the D2 approximation was used for the dispersion effects.^15^ For the reference binding energy of molecules with hydrogen bonds to the surrounding water, an empirical energy of 14 kcal/mol has been used. The only significant entropy effects for molecules without hydrogen bonds, such as NO, are those for the loss of translational entropy of free molecules taken from the gas phase. The effects are added to the computed enthalpic energies. The energies given in the text and tables are then approximate free energies. The errors using the present methodology have usually been found to be 3 kcal/mol or smaller.^3,5,11,12^ The programs used were Jaguar^14^ and Gaussian.^16^

The present calculations do not take into account the possibility that concentrations affect the results. It is assumed that the concentration of hydroxylamine is saturated. The cost of bringing hydroxylamine into the reaction is then about 14 kcal/mol, see above. For NO, there is no such problem, since NO is taken from the gas phase with only an entropy loss.

For the modeling of the enzyme active site, a cluster model^17^ was used with 130–140 atoms, see Figures 1 and 2. Apart from P460, the histidine bound to iron was included. The charge of the model for HAO is +1, while it is +2 for cyt-P460. Attempts were made to model the proton transfer pathway from P460, but it turned out that a significantly larger cluster was required, and such a modeling was therefore not pursued. The problem was that the carboxylate base needed a large region for stabilizing its negative charge. Surrounding the carboxylate with water molecules was tried but did not quite help. Cluster modeling requires that some atoms be fixed from the X-ray structures. The fixed atoms, about 10 in each case, are marked with a # in the SI.

An exact value for the redox potential used in vivo has been difficult to find. Instead, the step with the smallest exergonicity has been assumed to be exergonic by −3 kcal/mol, which leads to a 99% conversion of the reactant. A more exergonic value would lead to an unnecessary energy loss. This procedure leads automatically to the reference cost for creating a (H^+^,e^–^)-couple, which is the relevant value for the redox transition energy. There is one exception, where it is important to separate the energy for removing an electron and removing a proton, and that is described in the “The alternative N–N bond formation” subsection below. The reference value for a proton in water is 280 kcal/mol, at pH = 7.^18^ There are very large dielectric effects when the charge changes. It was decided to follow the general praxis of using a dielectric constant of 20.0 for this particular energy instead of 4.0.^19^ It should be emphasized that this is the only value in the present study where there are large dielectric effects since removing a (H^+^,e^–^)-couple does not change the charge of the model.

There is a general experience that for reactions taking milliseconds or more transition state theory very accurately takes care of dynamic effects. In TS theory, it is assumed that there is strict thermal equilibrium, and therefore, the energy levels follow a Boltzmann distribution. For reactions in microseconds and faster, detailed dynamic effects may play a role.

## III Results

**Figure 3:**
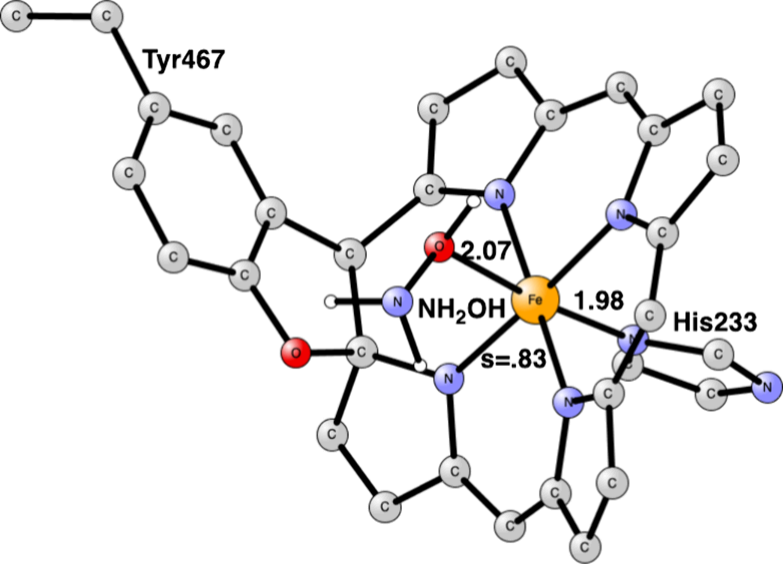
Hydroxylamine binding to the heme of HAO. Some atoms have been deleted for the sake of clarity. Distances are given in Å. The spin state is a doublet.

The model calculations will start with the mechanism for HAO. The first step is the binding of NH_2_OH to iron, see Figure 3. The binding is quite strong with a rather short bond distance of 2.07 Å to iron. The spin on iron is 0.83, indicating Fe(III) in agreement with experiments. The binding energy is calculated to be +17.2 kcal/mol, which includes a loss of −14 kcal/mol from the binding to the water medium. The spin state is a doublet. After that, the mechanism proceeds in three steps of removing (H^+^,e^–^)-couples, in which the protons are removed from NH_2_OH. The first step is the removal of the oxygen proton of NH_2_OH, which is the step with the smallest exergonicity and, therefore, is the one with the highest demand on the redox potential of the oxidant. It is assumed to be exergonic by −3 kcal/mol, see Section II. The product is NH_2_O, which is bound by its oxygen end to iron with a distance of 1.94 Å, see Figure S1. The spin on the oxygen is 0.48, while the spin on nitrogen is 0.34. The spin on iron is reduced from 0.83 to 0.08, indicating that Fe(II). Instead, there is a spin on P460 of −0.9. The spin state is a singlet.

In the next step, one of the hydrogens on nitrogen is removed. There is no spin on the NHO product, which binds with its oxygen end to iron with a distance of 2.00 Å; see Figure S2. Iron is back to Fe(III) with a spin of 0.81. There is no spin on NHO and P460. The step is exergonic by −8.6 kcal/mol. The spin state is a doublet.

**Figure 4:**
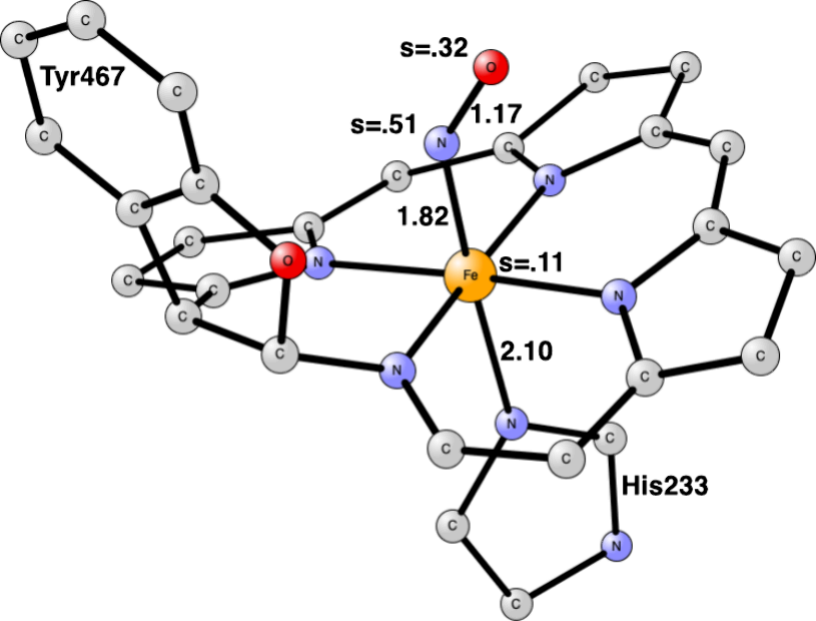
NO binding to the heme of HAO. Some atoms have been deleted for the sake of clarity. P460 has radical character. Distances are given in Å. The spin state is a singlet.

The final proton on the substrate leaves in a step leading to NO, initially bound by its oxygen end to iron. However, in a nearly barrierless reorientation, NO becomes bound with its nitrogen end to iron, which is much preferred; see Figure 4. NO is now bound by 10.9 kcal/mol, which includes the loss of translational entropy of 10.8 kcal/mol for free NO. The oxidation step is exergonic by −35.3 kcal/mol. The spin on oxygen is 0.32, and the one on nitrogen is 0.51. The spin on iron is 0.11 indicating Fe(II), and the one on P460 is −1.0. The spin state is a singlet.

In the final step, NO leaves, and another NH_2_OH substrate binds. That step is unproblematic since NH_2_OH is bound by 17.2 kcal/mol, much more than that of NO. The entire step from NHO to NO is very exergonic by −34.5 kcal/mol, which is much more exergonic than the other two transitions. That fact is important for avoiding the formation of an N–N bond, which is discussed below for an alternative N–N bond formation.

The above sequence of events leading from NH_2_OH to the final product NO appears to be straightforward, but there is a problem in avoiding the formation of the very stable N_2_O molecule, to be discussed below.

### Mechanism of cyt-P460

Initially, the mechanism for oxidation of NH_2_OH follows the same steps as in HAO. The binding of NH_2_OH is almost as strong as for HAO, 14.7 compared to 17.2 kcal/mol. Iron has a spin of 1.05, indicative of Fe(III). The structure is shown in Figure S3. Removing the first hydrogen is not the step with the smallest exergonicity, as it is for HAO. The exergonicity is −5.5 kcal/mol. The removal of the hydrogen on oxygen leads to a structure with a quite different electronic structure than the one in HAO. Iron remains Fe(III) with a spin of 1.05, and P460 has no spin. In HAO, iron was Fe(II) and P460 had a spin of −0.9. The spin on the oxygen of the substrate is −0.51, the same as that for the nitrogen. The state is a singlet.

Removing the second hydrogen in cyt-P460 leads to a structure with a spin on iron of 0.38, which is somewhere between those of Fe(II) and Fe(III). P460 has a spin of 0.9. The spin on the nitrogen of the substrate is −0.33. Since this step is the one with the smallest exergonicity, the exergonicity is assumed to be −3.0 kcal/mol, see Section II.

When the third hydrogen is removed from the substrate, a structure with NO bound to iron is reached. The wave function is very similar to the one in HAO. Iron is in a Fe(II) state with a spin of 0.20, while the spin on P460 is −1.0. On NO, the spins are 0.50 on nitrogen and 0.30 on oxygen. NO is bound by 13.5 kcal/mol, compared to 10.9 kcal/mol in HAO. The (H^+^,e^–^) removal is exergonic by −31.0 kcal/mol, compared to −35.3 kcal/mol for HAO.

### N–N Bond Formation

The two competing reactions for the P460 enzymes are NO release and N_2_O formation and release. The first one is the exclusive product for HAO, and the second one is the exclusive product for cyt-P460.

**Figure 5:**
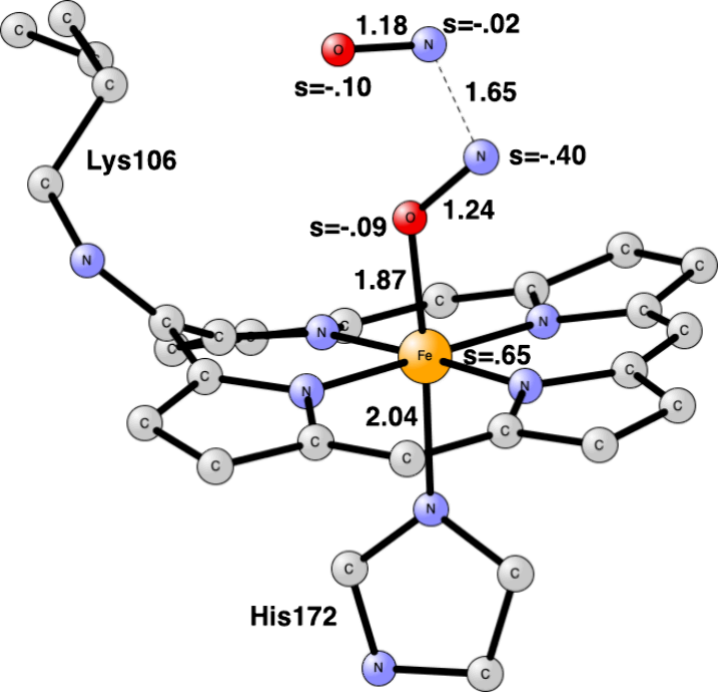
Transition state for the N–N bond formation in cyt-P460. Some atoms have been deleted for clarity. P460 has radical character. Distances are given in Å.

For forming an N–N bond, a second NH_2_OH substrate should be needed. The first attempts were made to directly form a bond between the NO and the added NH_2_OH, but they were unsuccessful. It became clear that there are too many hydrogens for that type of bond formation. Instead, another NO was brought in from the gas phase at the cost of losing the translational entropy of +10.8 kcal/mol. Starting with cyt-P460, the optimized TS for N–N bond formation is shown in Figure 5. The TS was surprisingly easy to find. The NO bound to iron first had to be rotated to bind with the oxygen end to iron, which led to an increase in the spin to 1.0. When the N–N distance is shortened in steps, the incoming NO loses most of its spin as the TS is approached, and the spin on the NO bound to iron goes from 0.98 to 0.49 at the TS. Iron obtains a spin of 0.65, which is somewhere between Fe(II) and Fe(III), while P460 gets a spin of 0.9. All of these changes occur automatically in the geometry optimization. At the TS, the N–N bond is 1.65 Å, and the Fe–O bond is 1.87 Å. The computed barrier is +16.6 kcal/mol, which includes a loss of translational entropy of 10.8 kcal/mol. This means that the N–N bond formation is feasible for cyt-P460, which is in line with experiments. It is endergonic by +7.1 kcal/mol. The product N_2_O_2_ structure is shown in Figure S4.

**Figure 6:**
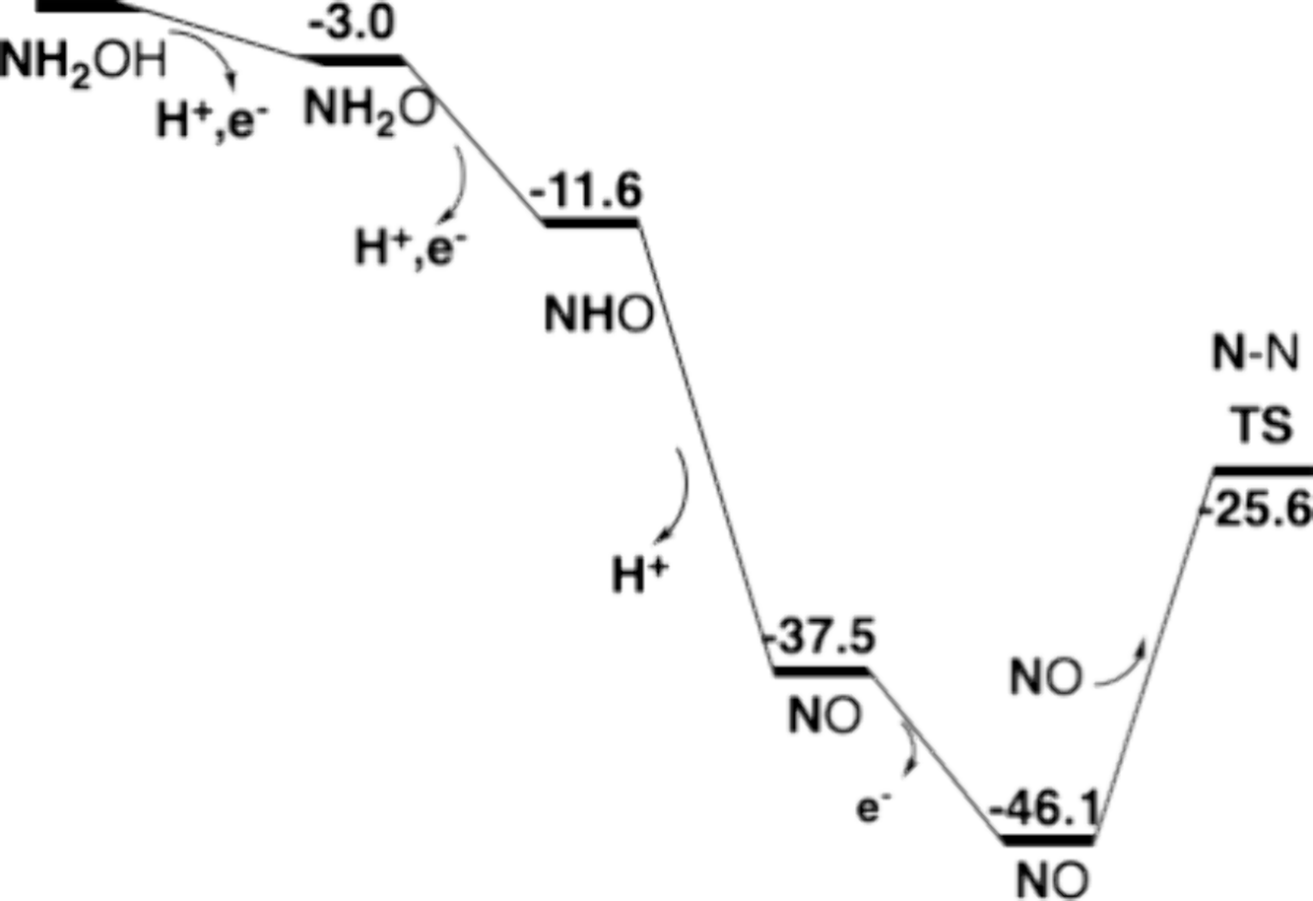
Energetics for oxidation of hydroxylamine to NO by HAO. Energies are in kcal/mol.

**Figure 7:**
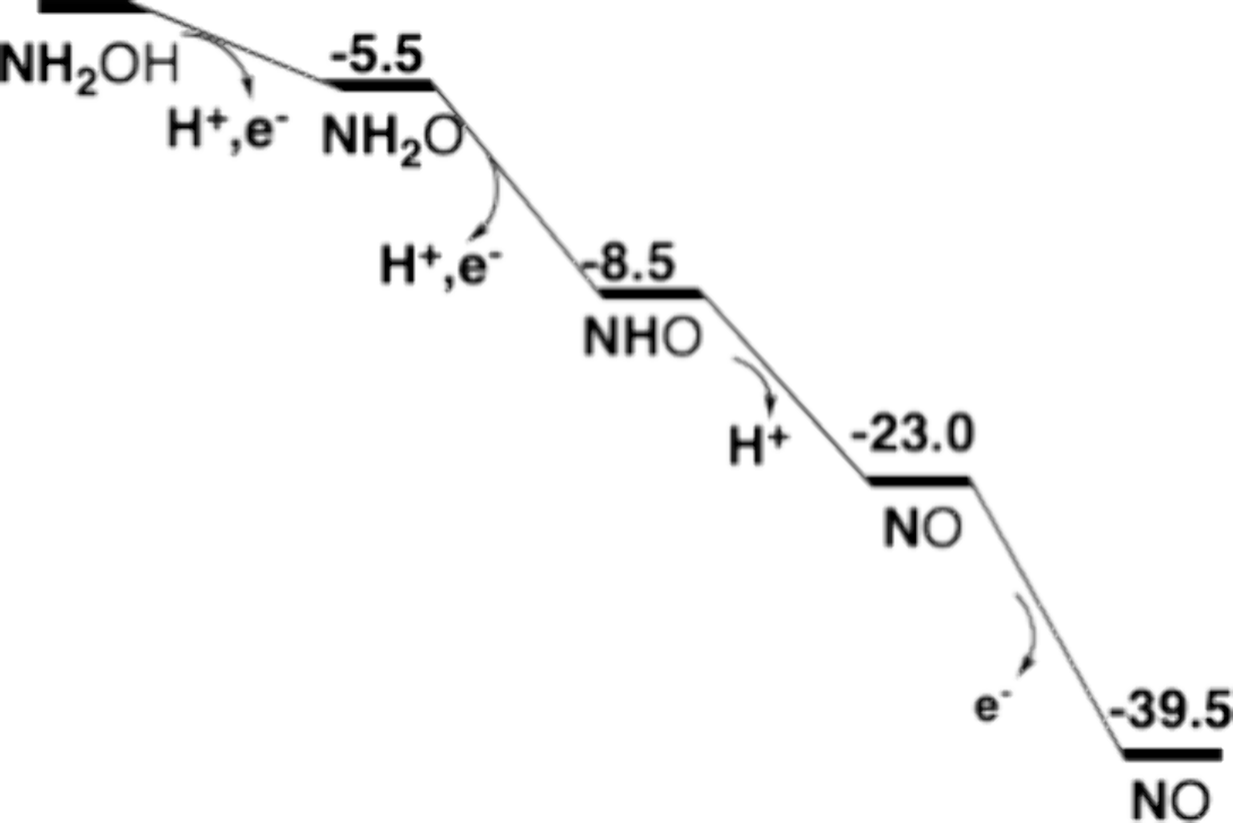
Energetics for oxidation of hydroxylamine by cyt-P460, first part. Energies in kcal/mol.

The same mechanism for HAO leads to somewhat different energetic results. The structure of the TS is quite similar to an N–N distance of 1.60 Å and an Fe–O bond of 1.92 Å; see Figure S5. The spin distribution is also similar but with a slightly higher spin on iron, 0.80 compared to 0.65. However, these rather small differences led to a higher barrier for N–N bond formation with 20.5 kcal/mol, compared to 16.6 kcal/mol for cyt-P460, which could explain why HAO does not lead to N_2_O production but only to NO release. The energetics for HAO are given in Figure 6, and the first part of the oxidation for cyt-P460 is shown in Figure 7.

**Figure 8:**
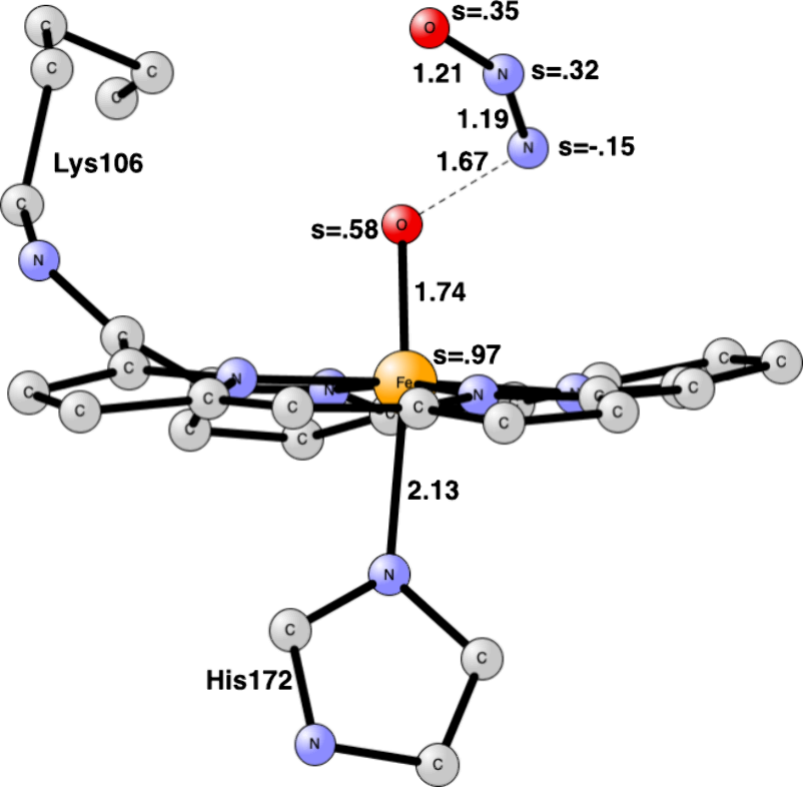
Transition state for the O–N bond cleavage leading to the formation of N_2_O in cyt-P460. Some atoms have been deleted for clarity. P460 has radical character. Distances are given in Å.

After N–N bond formation in cyt-P460, the next step is the cleavage of the O–N bond for the release of N_2_O. The optimized TS is shown in Figure 8. The N–O distance is 1.67 Å at the TS, and there is a significant shortening of the Fe–O distance from 1.87 Å for the TS in Figure 5 to 1.74 Å in Figure 8. The spin on iron is 0.97, indicating a rather clean Fe(III), and the spin on P460 is −0.8. The N_2_O part has a significant spin of 0.82 and the oxo-group 0.58. The barrier is very small with only 4.0 kcal/mol and the large exergonicity is −35.4 kcal/mol. After the release, N_2_O has no spin, the oxo group is 0.86, iron is 1.22, and P460 is −1.0. From the starting point with NO bound to iron to the N_2_O product, the reaction is exergonic by −28.3 kcal/mol.

**Figure 9:**
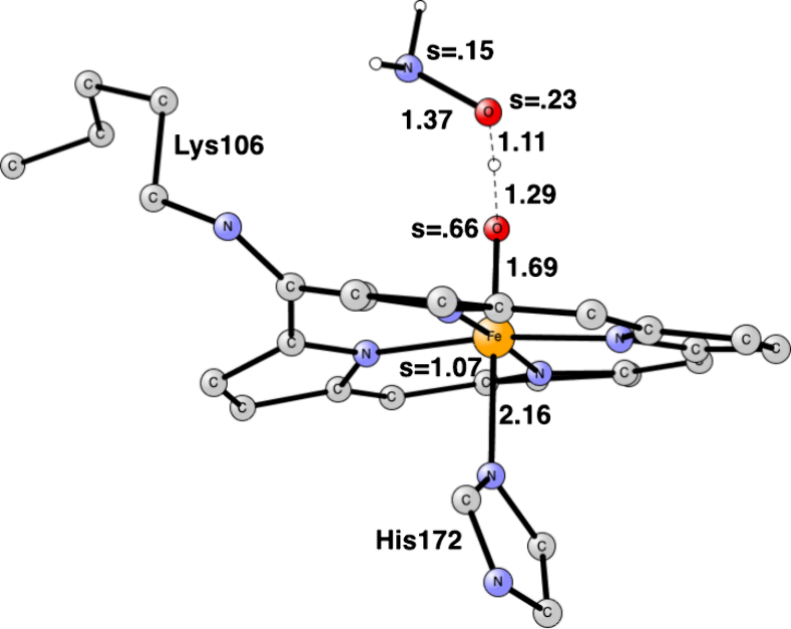
TS structure for the abstraction of hydrogen from NH_2_OH in cyt-P460. Some atoms have been deleted for clarity. Distances are given in Å. P460 has a radical character.

The O–N bond in cyt-P460 leads to the formation of an Fe(III)-oxo group. The oxo group gets a spin of 0.94, iron a spin of 1.15, and P460 a spin of −1.0. The oxo must be removed if the reaction sequence above should be repeated. It turns out that the oxo can easily abstract a hydrogen from an incoming NH_2_OH, which becomes bound with a hydrogen bond to the oxo. The binding energy is 3.0 kcal/mol, which includes the loss of 14 kcal/mol from taking NH_2_OH from the water surrounding. The TS for hydrogen abstraction from NH_2_OH by the oxo is shown in Figure 9. The barrier is only 3.7 kcal/mol. The reaction is exergonic by −11.4 kcal/mol. The resulting NH_2_O has one spin.

**Figure 10:**
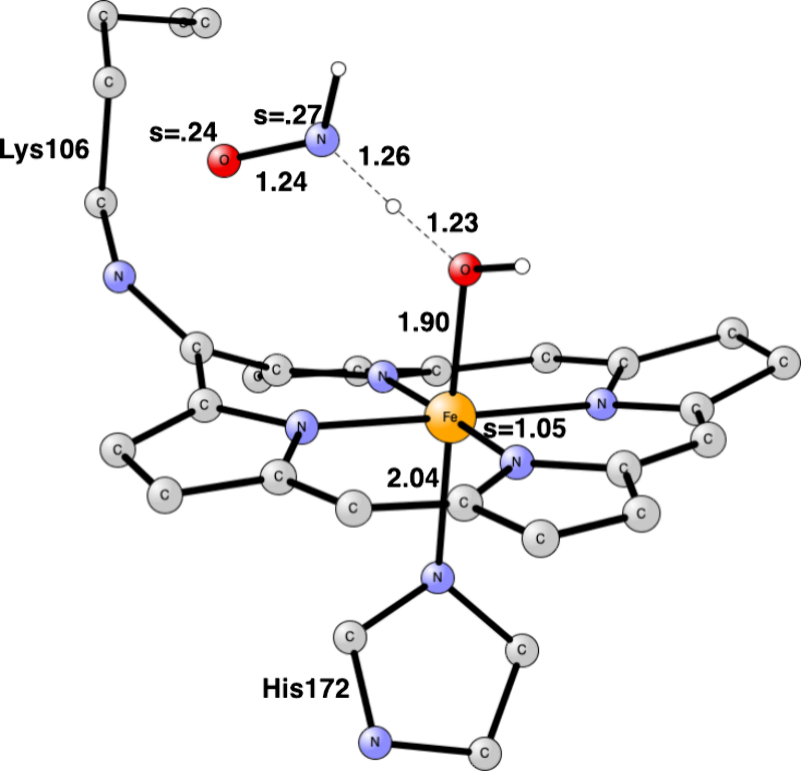
TS structure for the abstraction of hydrogen from NH_2_O in cyt-P460. Some atoms have been deleted for clarity. Distances are given in Å.

The product of the hydrogen abstraction is NH_2_O which is bound by 1.3 kcal/mol to Fe–OH compared to NH_2_O in the water medium. The TS for hydrogen abstraction from NH_2_O is shown in Figure 10. With all corrections included, the barrier is essentially zero. The exergonicity is −4.6 kcal/mol. The resulting NHO has no spin. The product H_2_O is bound by 10.0 kcal/mol to Fe(III)-P460, see Figure S6. An interesting aspect is that as water is removed from iron in steps, the wave function continuously changes character to triplet Fe(II) with a P460 radical. That is not the case for HAO, where the wave function remains Fe(III) with a nonradical P460.

**Figure 11:**
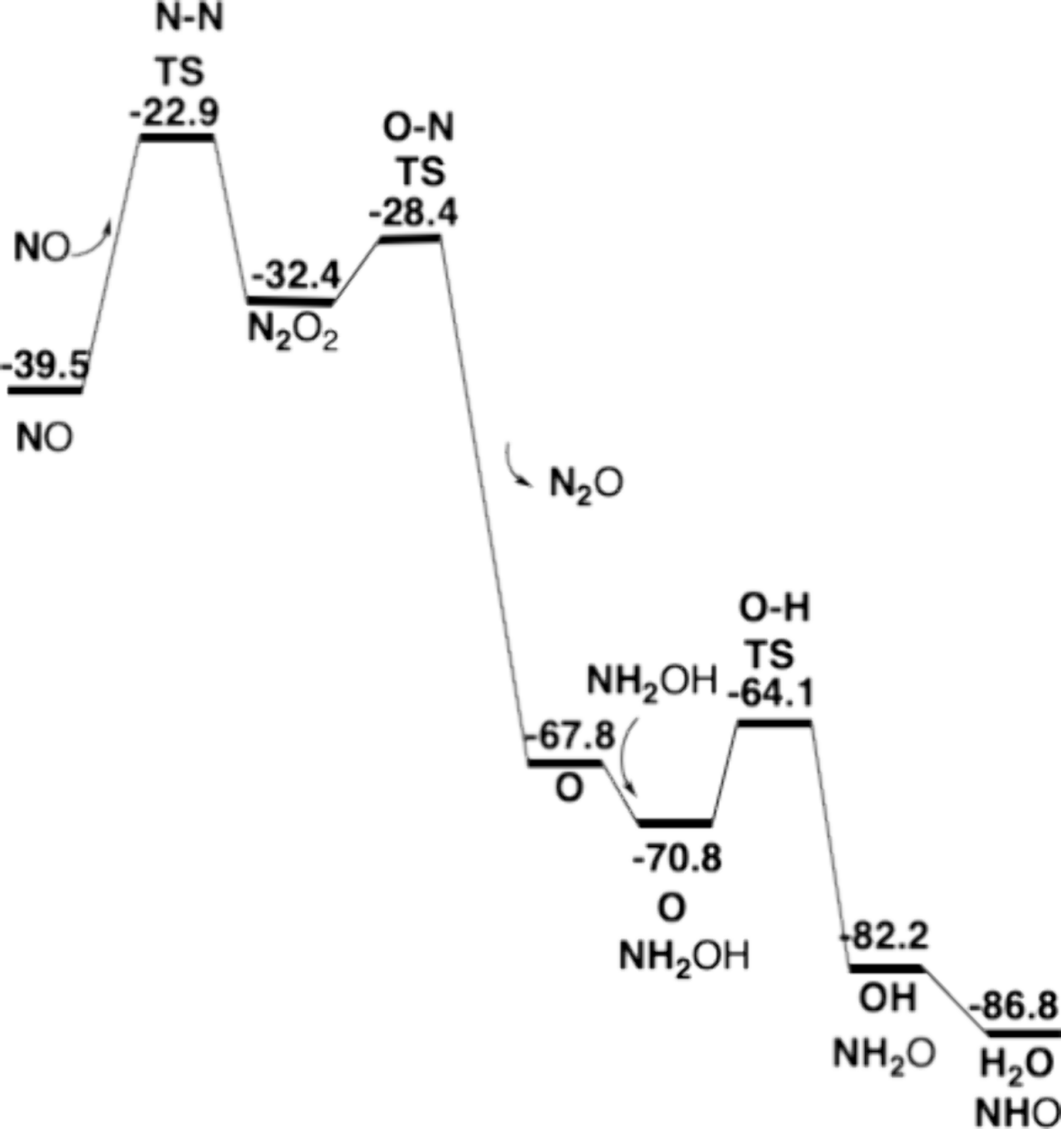
Energetics for oxidation of hydroxylamine by cyt-P460, second part. Energies in kcal/mol.

It should be noted that the second round of NH_2_OH oxidation in cyt-P460 will not be the same as the first round described above. After water is removed, the second round will start at the point where NHO is reached. The energetics for the second part of the oxidation of hydroxylamine to form N_2_O is shown in Figure 11.

### Effect of the Lysine

To study the effect of covalently bound Lys106 in cyt-P460, it was replaced by a hydrogen. The first test was for the binding of NO to iron. There is a large increase in the binding of NO by 8.8 kcal/mol with the attached Lys106. The most notable effect occurs for naked P460, where the spin on iron changes from 1.32 without lysine to 2.00 with lysine, which implies much more Fe(III) character without Lys106. When NO becomes bound, the wave functions with and without Lys106 are nearly identical.

There are also notable changes in the critical step of the N–N bond formation. The barrier decreases from 22.5 to 16.6 kcal/mol as Lys108 is attached, which is undoubtedly the main reason for attaching Lys108. For the structures of the two TS, see Figure 5 and Figure S7, the main effect is a lengthening of the N–N distance from 1.36 to 1.65 Å when Lys108 is attached. Without Lys108, the incoming NO has to get substantially closer to the bound NO for forming the N–N bond than with the lysine. There are no changes around the heme by the attachment. For example, the Fe-NHis bond distances are almost exactly the same. The differences in the wave functions with and without Lys108 are rather subtle. The main effect is on the spin of iron, which goes from 1.02 without to 0.65 with Lys108, indicating more Fe(II) character with lysine.

### Effect of the Tyrosine

The effect of Tyr467 on HAO was investigated by removing it, just as for Lys106 for cyt-P460. The step with the smallest exergonicity in HAO is the removal of the first proton from NH_2_OH. Compared to the case with Tyr467, that led to a reduction of the exergonicity by 6.8 kcal/mol. The result gives a clear reason for why Tyr467 has become covalently bound in HAO. The dominant effect on the wave function is an increased spin on iron from 0.83 to 1.04 when Tyr467 is removed, which indicates an increased Fe(III) character, which, in turn, leads to a stronger Fe–O bond strength. For the product NH_2_O, there is no similar effect.

### Alternative N–N Bond Formation

The redox transitions discussed above all used the proton-coupled redox potential. There is one situation where this becomes problematic. If NHO is formed after removing a (H^+^,e^–^)-couple from NH_2_O, the resulting NHO molecule bound to iron will form an N–N bond with an external NO molecule, with a quite small barrier. That is not a problem for cyt-P460, since it should form an N–N bond, but it is a problem for HAO, which should not do it. As noted above, the third redox transition has a very large exergonicity, much larger than that for the two first ones. That indicates a possibility for the proton to leave the previous transition. Using the reference value of 280 kcal/mol and a dielectric constant of 20.0, see Section II, the energy for removing only the proton was calculated. The result was that the proton was very easy to remove. In other words, the second redox transition is not one with the removal of a (H^+^,e^–^)-couple but one with a removal of (2H^+^,1e^–^), leading directly to an NO molecule bound to iron. As indicated above in Section II, the energy for removing just a proton has a higher uncertainty than the removal of a (H^+^,e^–^)-couple due to long-range effects. However, in the present case, the exact value is not important. What is important is that the removal is exergonic. The exergonicity was calculated to be −14.5 kcal/mol, so the margin is large. When the second proton was removed, the N–N bond formation at that stage was calculated to be very endergonic by +16.6 kcal/mol and would not occur, neither for HAO nor for cyt-P460. The high endergonicity shows that an additional oxidation is definitely needed for forming the N–N bond, see above. The final redox transition would then be one where only an electron is removed, and where N–N bond formation would occur for cyt-P460 but not for HAO, see above.

## IV Conclusions

The oxidation of hydroxylamine has been investigated for the two enzymes that are able to perform the oxidation, HAO and cyt-P460. Both use the unusual P460-heme as a cofactor. HAO has a covalently bound tyrosine, and cyt-P460 has a covalently bound lysine. After three reductions, both have a structure with NO bound to iron. While cyt-P460 can proceed to form a N–N bond with an incoming NO with a feasible barrier of 16.6 kcal/mol, HAO has a prohibitively high barrier of 20.5 kcal/mol. HAO avoids N–N bond formation at an earlier stage by having a (2H^+^,1e^–^) release from NH_2_O in the second oxidation step.

An important factor in catalysis is that two states of P460 with different electronic structures are easily accessible. In one of these states, iron is Fe(III) with no spin on P460, while in the other one, iron is Fe(II) with a P460 with high spin.

After N–N bond formation in cyt-P460, which is endergonic by +7.1 kcal/mol, the next step is to break the O–N bond, which is very exergonic, leading to an overall exergonic formation of N_2_O. The release of N_2_O leads to the remaining oxo on the heme. The oxo can abstract two protons from the next NH_2_OH, leading to a second round of oxidations different from that for the first one. The calculations agree with the finding that the anaerobic oxidation of hydroxylamine in HAO leads to the formation of NO, while the one in cyt-P460 leads to N_2_O.^6^

The effect of the covalently linked tyrosine in HAO was investigated by removing the tyrosine. For the step with the smallest exergonicity, which is the first oxidation, the attached tyrosine increases the exergonicity by 6.8 kcal/mol. The effect of the covalently linked lysine was investigated in a similar way. The barrier for the rate-limiting step of N–N bond formation was reduced by 5.9 kcal/mol by the attachment. These results give clear explanations for why P460 has unusual attachments to the two enzymes.

## Supporting Information Available

Coordinates for the structures discussed in the text together with additional figures (PDF)
