# Supplementary material for: Nitrification Mechanisms for the P460 Enzymes
Source: J Phys Chem B. 2024 Dec 18;129(1):111–6. doi: 10.1021/acs.jpcb.4c06537 (PMC11726666; doi:10.1021/acs.jpcb.4c06537)
Supplement: Supplementary file 1 — jp4c06537_si_001.pdf [file jp4c06537_si_001.pdf]

Supporting information:

The Nitrification Mechanisms for the P460 Enzymes.

Per E. M. Siegbahn*

Department of Organic Chemistry, Arrhenius Laboratory, Stockholm University,

SE-106 91, Stockholm, Sweden. Email:per.siegbahn@su.se

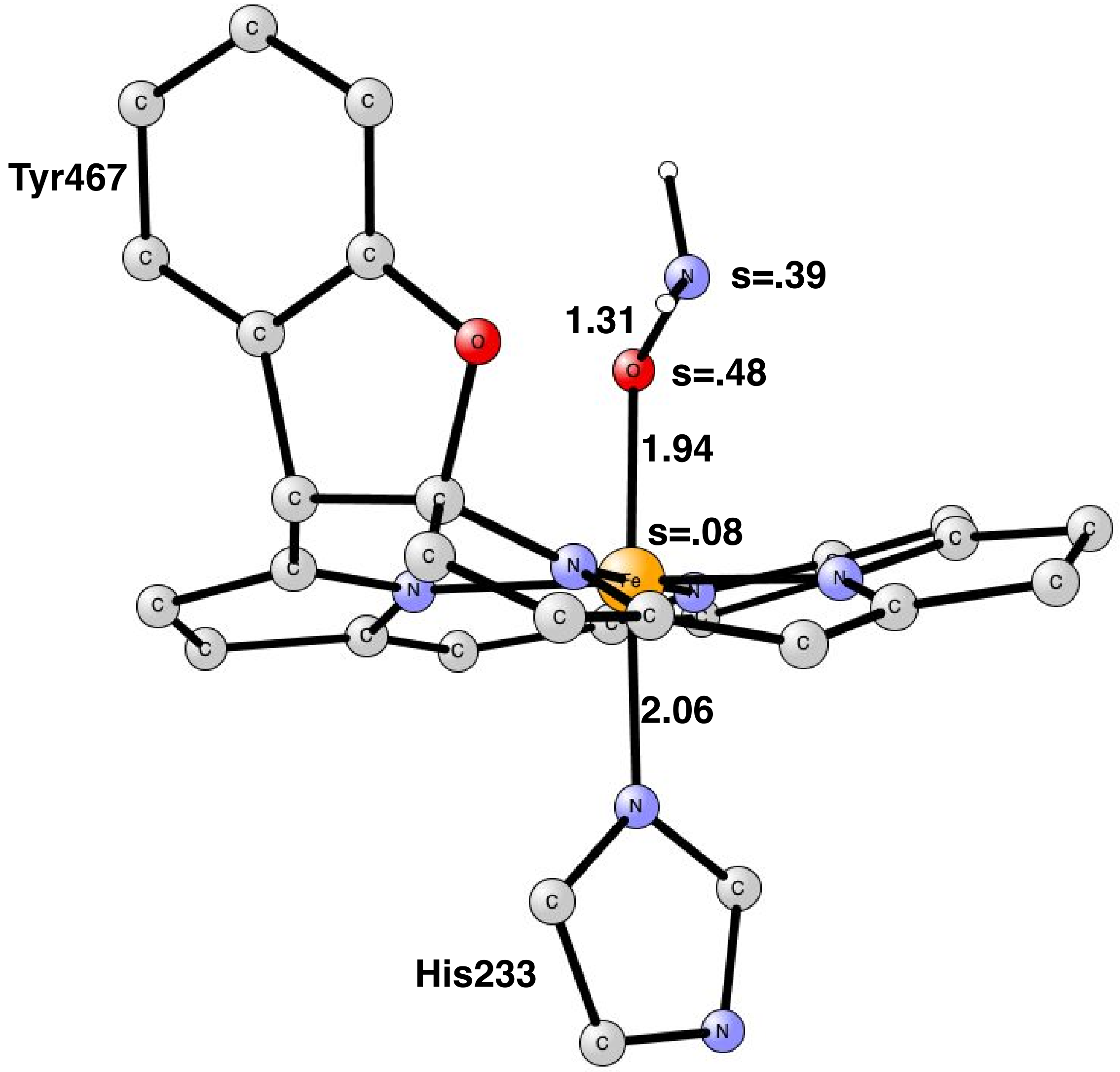

Figure S1. ONH_2 binding to the heme of HAO. Some atoms have been deleted for clarity. Distances are given in Å. The spin on P460 is -1. The spin-state is a singlet.

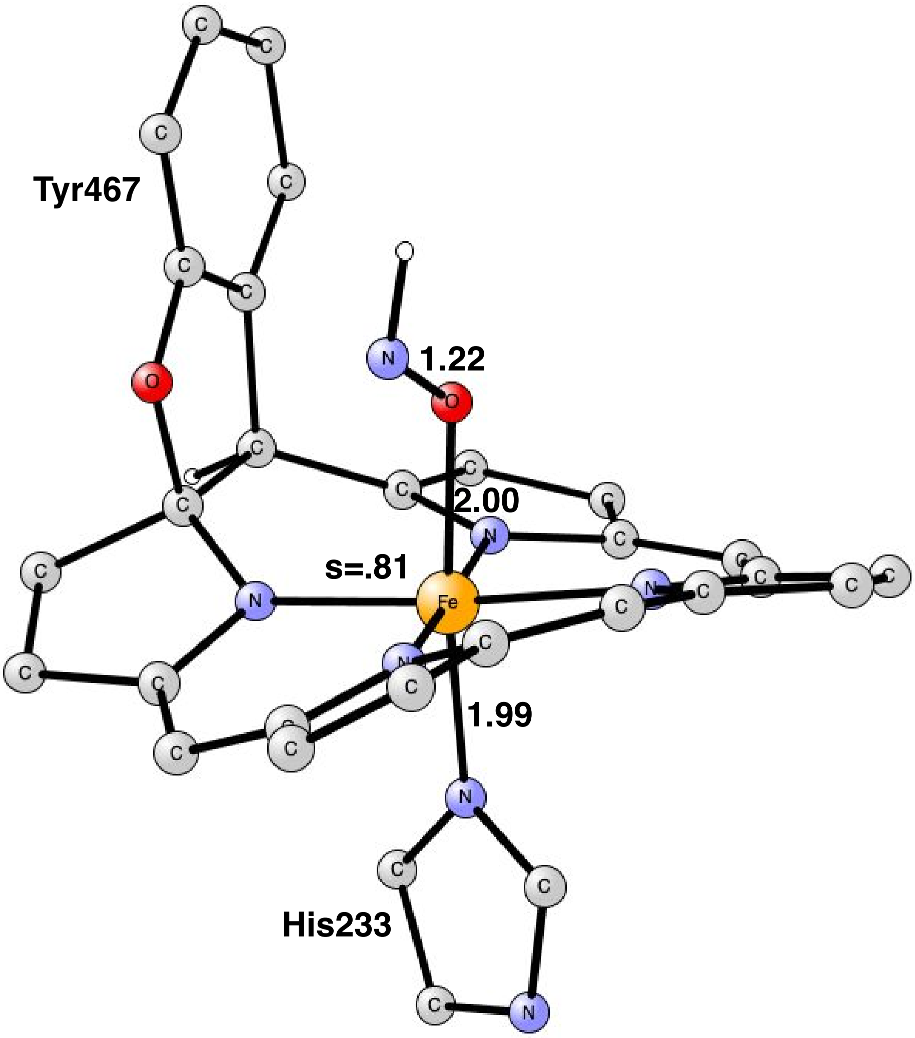

Figure S2. NHO binding to the heme of HAO. Some atoms have been deleted for clarity. Distances are given in Å. The spin-state is a doublet.

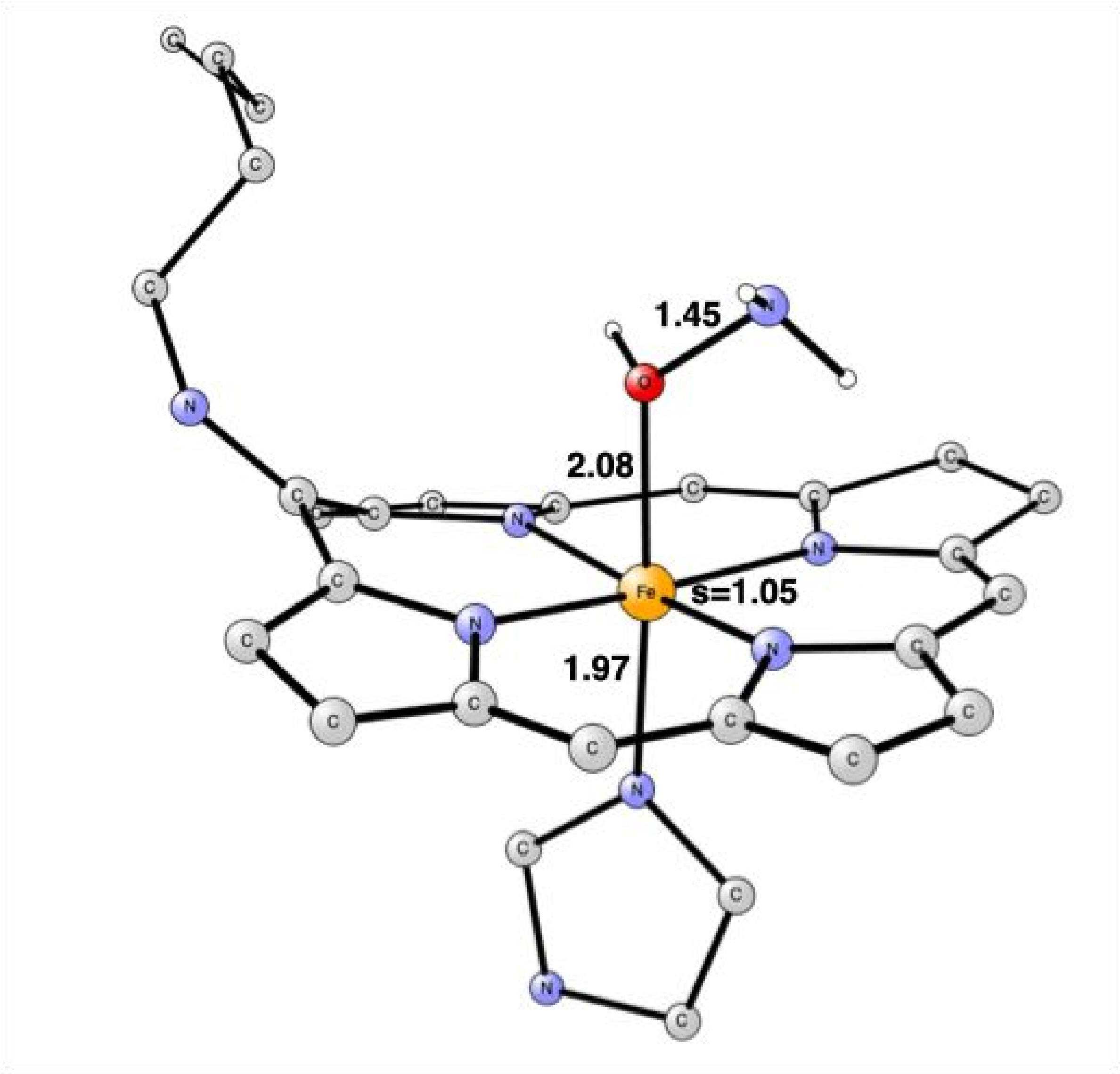

Figure S3. NH_2OH binding to the heme of cyt-P460. Some atoms have been deleted for clarity. Distances are given in Å. The spin-state is a doublet.

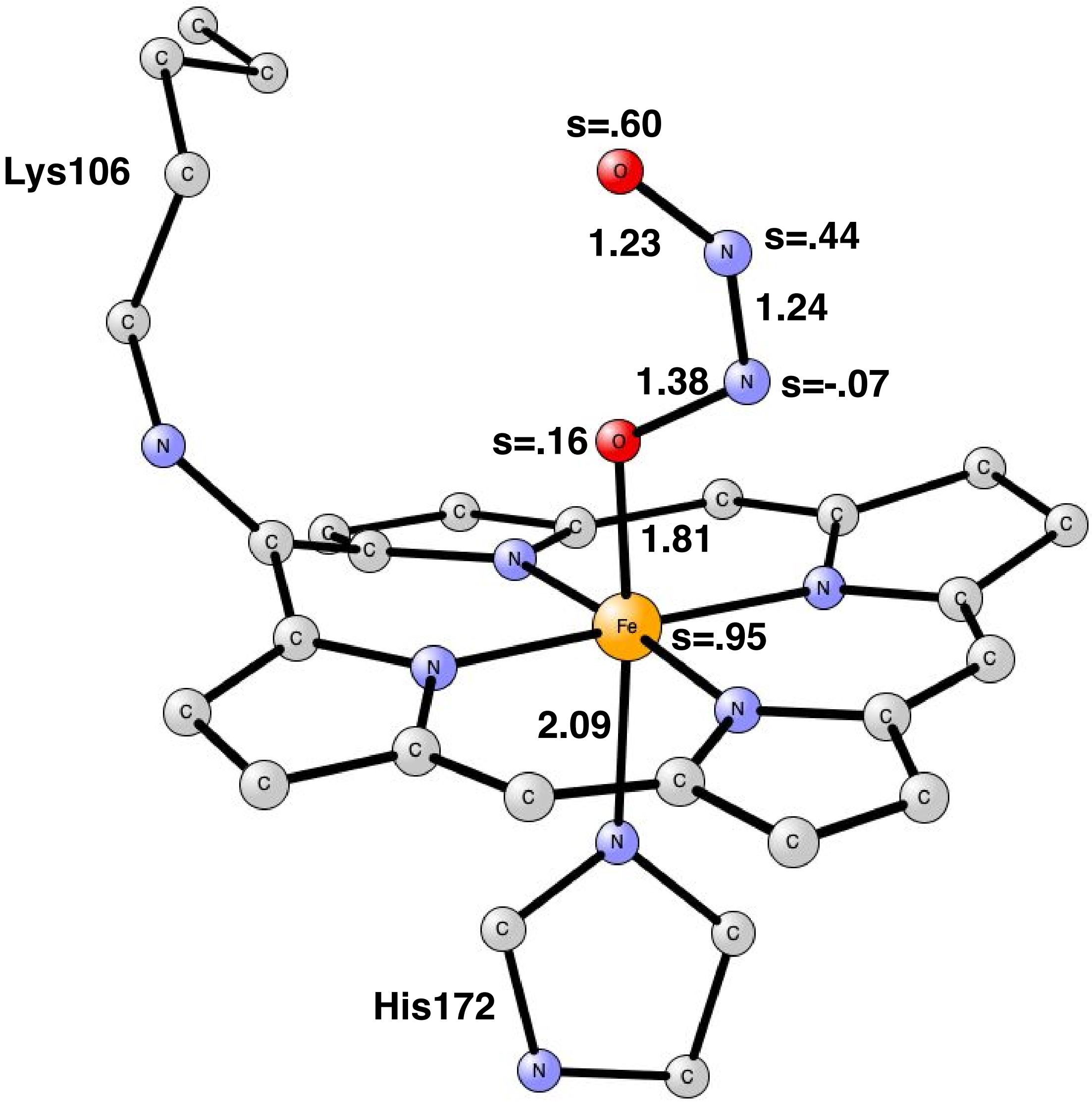

Figure S4. N_2O_2 binding to the heme of cyt-P460. Some atoms have been deleted for clarity. Distances are given in Å. The spin on P460 is -1, and the spin-state is a doublet.

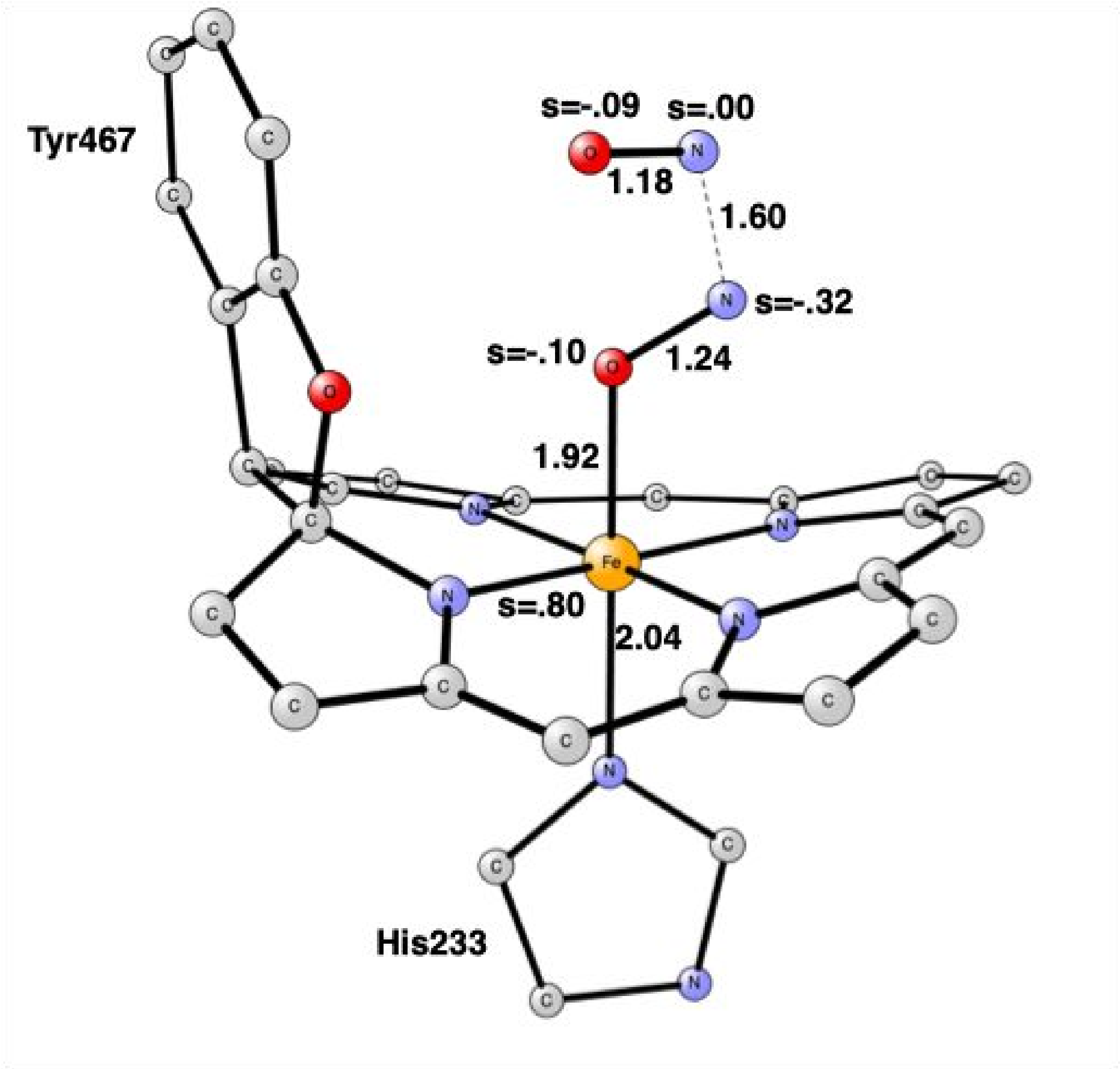

Figure S5. Transition state for N-N bond formation in HAO. Some atoms have been deleted for clarity. P460 has some radical character. Distances are given in Å. The spin-state is a doublet.

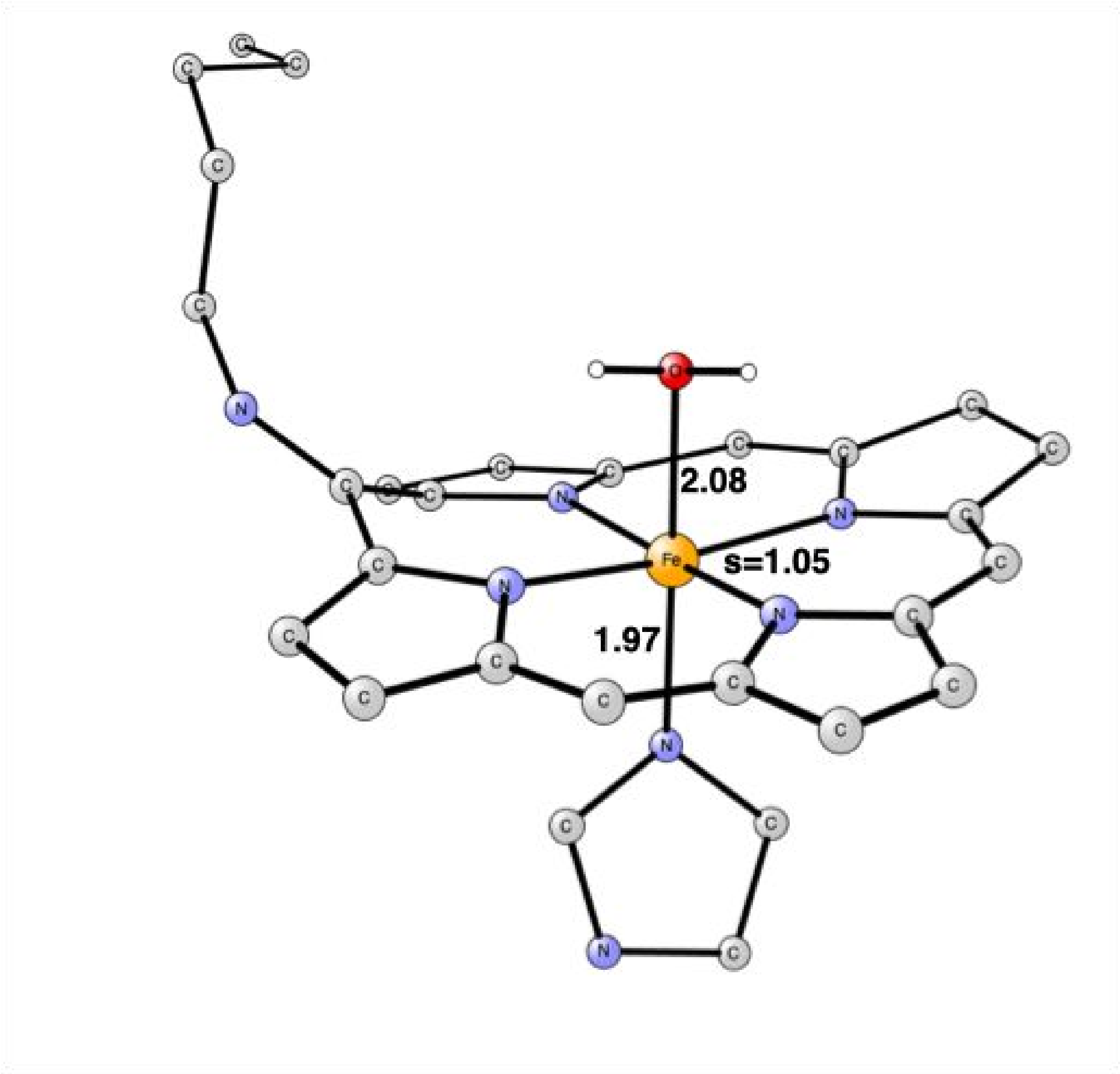

Figure S6. H_2O binding to the heme of cyt-P460. Some atoms have been deleted for clarity. Distances are given in Å. The spin-state is a doublet.

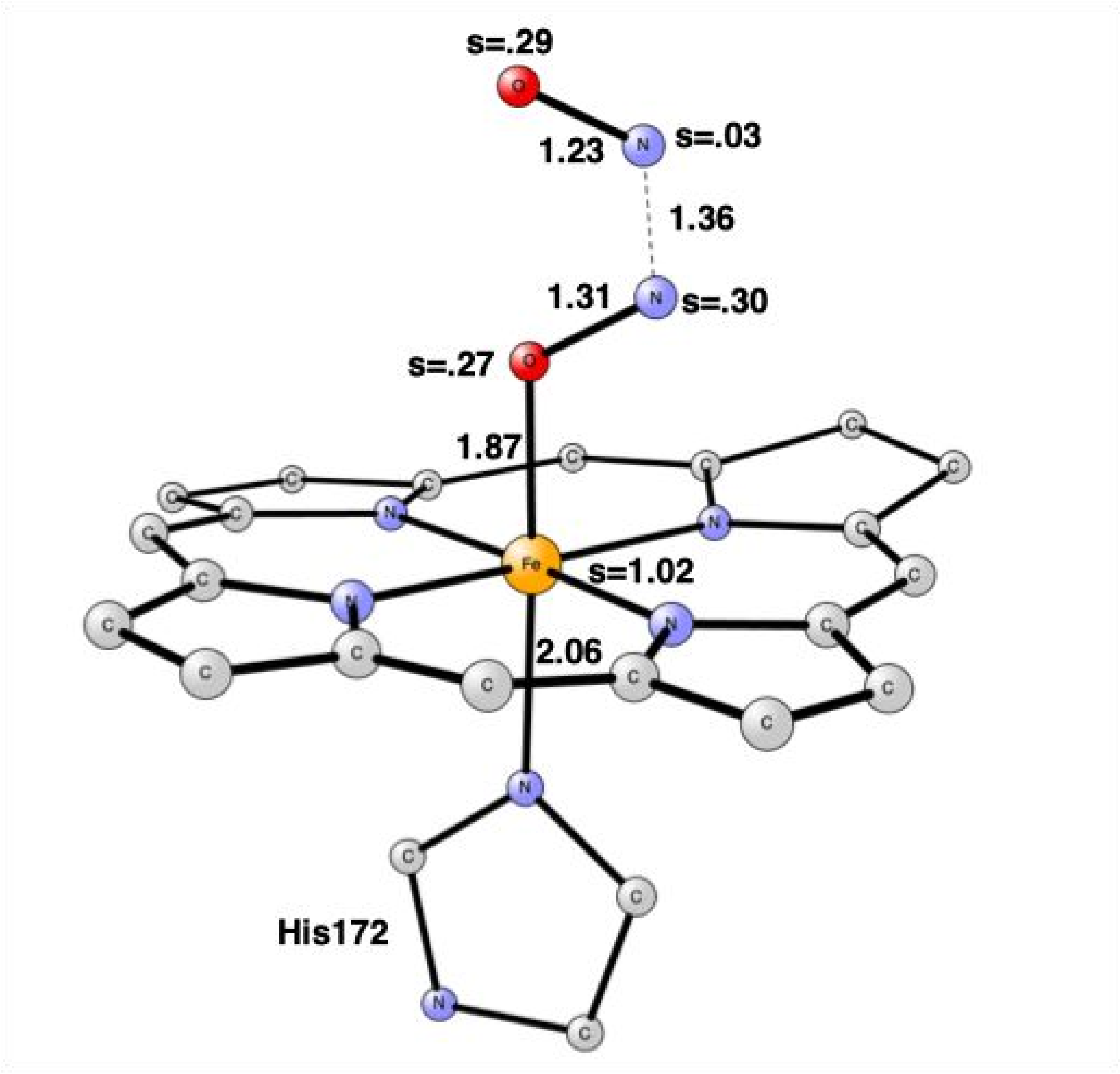

Figure S7. Transition state for N-N bond formation in cyt-P460 without Lys106. Some atoms have been deleted for clarity. P460 has radical character. Distances are given in Å.

The structure in **Figure 3.** # means fixed atom

Energies: E= -3883.381241, solv = -0.077450, disp = -184.36 Z_0 = 703.63

Fe1	10.0679130893	48.9430341708	25.5735693628
N2	8.1330241668	48.6214229034	25.9155853467
N3	9.9651105620	50.3891085139	26.8134019108

N4	12.0493870928	49.2704103065	25.2576249636
N5	10.1619542696	47.2753127397	24.3953669565
C6	7.4697730229	47.4167777758	25.7393874109
O7	2.4580000368#	47.4399990106#	26.0699999037#
C8	8.8740228517	51.1760988284	27.0594739162
C9	12.8781764632	50.0907367431	25.9241431373
C10	11.1596256680	46.9316736572	23.4915945754
O11	8.8704008587	40.9628015649	21.9699528183
C12	6.1015255509	47.5424847767	26.2402592141
O13	3.3258096951	48.2150607036	24.1687739348
C14	9.2477794892	52.3776280486	27.8516220413
C15	14.1832963049	50.1853881071	25.2861090214
C16	10.7961523306	45.7142011360	22.7838844092
O17	7.2990000340#	41.4389990003#	23.5170000150#
C18	5.8877288437	48.8628229468	26.5669537142
C19	10.5595378954	52.2212497021	28.1704418742
C20	14.1126582617	49.3214985408	24.1921929353
C21	9.5605799939	45.3396384401	23.3100366462
C22	7.2112635440	49.5085020562	26.3873173819
C23	11.0251680922	50.8344966523	27.7040845291
C24	12.7839141372	48.7300934937	24.1933206499
C25	9.1917982132	46.3200267423	24.2949606011
C26	5.1814639896	46.3555566582	26.3300313209
C27	11.4366444679	53.0160045935	29.0754251574
C28	15.1893656422	48.9625541152	23.2026065106
C29	8.7493786495	44.1433440466	22.8884361204
C30	4.2656715142	46.1794309269	25.0918501541
C31	10.6800002032#	53.7659989394#	30.1639996557#
C32	16.5419997894#	48.6100011555#	23.8370000512#
C33	9.2443285960	42.8066924210	23.5022257249
C34	3.3090184370	47.3525410181	25.0102045291
C35	8.4946625073	41.6330466055	22.9046469680
C36	7.9663205959	46.3552504705	25.0192851007
C37	7.5604542292	50.8146769292	26.7732428023
C38	12.5407593176	50.6881873138	27.2669805464
C39	12.3539513494	47.6726386209	23.3952900685
C40	4.5840005900#	49.5429986732#	27.0259989544#
C41	8.2430003967#	53.4420003578#	28.2979997292#
C42	15.4039996829#	51.0260008085#	25.7099991713#
C43	11.5399995934#	44.9049994928#	21.7090010378#
C44	12.9969999527#	54.0449979543#	25.3990003696#
C45	12.0222820872	54.4907352568	26.4971241402
S46	12.6861364971	54.1298818172	28.1876205490
N47	13.2252797155	52.4095702440	22.4294681528
C48	13.2833772892	51.8030892543	21.0947556547
C49	11.8509412924	51.7440830710	20.5366393793
C50	13.9247594896	50.3988412843	21.0370318684
S51	15.5359180487	50.2835112052	21.9211421058
N52	11.2816350063	53.0877800729	20.5159851659
C53	9.8353329829	53.2001877907	20.5383859435

C54	9.1603840163	53.1442372011	21.9437016187
C55	9.2136579734	51.8336750127	22.6643016656
N56	8.5060105029	50.7191880888	22.2338933047
C57	9.8582753917	51.4130999900	23.8007739808
C58	8.7307952681	49.6953019318	23.0837665774
N59	9.5502883565	50.0866120330	24.0489843885
C60	16.9891349748#	48.9429349515#	31.0169701179#
C61	16.1290934574	48.1041720586	30.0459738415
C62	14.7232144402	48.6294615528	29.7915731222
C63	13.6722783432	48.3364236071	30.6760186551
C64	14.4355096330	49.3977832430	28.6471160319
C65	12.3635036801	48.7809549510	30.4510501522
C66	13.1393413725	49.8498523822	28.4049162511
C67	12.1261066757	49.5333590826	29.3032495242
O68	10.8995921139	50.0170698131	28.9421930102
H69	17.0348803482	49.4818133721	24.2714450696
H70	17.2115248620	48.1931843160	23.0789268029
H71	16.4028261746	47.8628273964	24.6255826079
H72	16.6551447171	48.0236993049	29.0862599025
H73	16.0565689004	47.0811363600	30.4368836392
H74	16.5168605047	49.0143979217	32.0030105705
H75	17.1318644413	49.9620276262	30.6406288577
H76	15.2368923932	49.6241781833	27.9531507159
H77	11.5641319592	48.5627897980	31.1527519346
H78	13.8757228545	47.7398964422	31.5619047004
H79	5.7745468025	45.4455326929	26.4655725015
H80	4.5666394770	46.4425361617	27.2336240539
H81	7.6932969085	44.2795469896	23.1436445562
H82	8.7871314393	44.0515238241	21.7955712425
H83	4.8516342713	46.1468508361	24.1711121616
H84	3.7081397285	45.2369459454	25.1752680070
H85	10.3042912482	42.6614514656	23.2821429327
H86	9.1100974972	42.8233383274	24.5885562259
H87	3.9164045619	48.8228448179	27.4993599339
H88	4.0543391712	49.9702320245	26.1689085482
H89	4.7935754518	50.3436527779	27.7424210957
H90	12.2811332724	45.5127876387	21.1809736295
H91	10.8326470540	44.5192286072	20.9694625408
H92	12.0644551521	44.0502066415	22.1508840734
H93	9.5468051042	54.1639860056	20.0985631618
H94	9.4107381661	52.4296032666	19.8810846003
H95	11.6938082197	53.6058861690	21.2910066848
H96	14.1745290411	52.5734169271	22.7612642772
H97	9.9958866377	53.0872775842	30.6859988818
H98	10.1165703117	54.6158904787	29.7779552111
H99	11.3872404132	54.1621380720	30.8996060968
H100	7.8844827790	53.2431914472	29.3132464515
H101	7.3769635831	53.4596957624	27.6307733786
H102	8.6851647140	54.4387183894	28.2796134512
H103	6.7660225190	51.4926125233	27.0571979634

H104	7.3259604857	45.4871111890	24.9125001813
H105	6.8733512346	40.6904930908	23.0556924892
H106	2.4723754700	46.6233214607	26.5954909979
H107	13.8659599826	52.4862148293	20.4650002512
H108	13.2472361134	49.6682692292	21.4923730556
H109	14.0864127378	50.0974560410	19.9967797509
H110	9.6290387772	53.9017978394	22.5819623480
H111	8.1092190672	53.4495764934	21.8343207222
H112	10.5121958264	51.9705329651	24.4507934984
H113	7.9083839850	50.6768481542	21.4191256075
H114	8.2890404915	48.7168584788	22.9890833982
H115	13.9776693277	54.5129523065	25.5339871573
H116	12.6236136189	54.3280932392	24.4084872017
H117	13.1370312462	52.9611995316	25.4073480685
H118	11.8445745890	55.5685649271	26.4421414165
H119	11.0532671543	53.9922442765	26.3965594392
H120	11.8650027779	51.3292266502	19.5188484056
H121	11.2790228811	51.0329161475	21.1617552065
H122	15.1294802120	51.7869149848	26.4457144832
H123	15.8263239423	51.5320962110	24.8362712448
H124	16.1905609399	50.4037939420	26.1473001673
H125	13.0572273661	47.3155466119	22.6497293830
H126	12.1139221481	52.3105702343	29.5684577419
H127	14.8656583003	48.0909534448	22.6293266539
H128	12.9830675014	51.6900747270	27.3047896335
H129	12.8198338231	51.7407827614	23.0851405700
H130	17.9753614652	48.4844962712	31.1446587276
H131	9.3284882535	48.1148167133	28.2869209602
N132	9.8855285334	47.2663383932	28.1753747043
H133	10.5605268790	47.3019234439	28.9441023811
O134	10.7334071627	47.6186989757	27.0342101209
H135	10.7517562786	46.8012506649	26.5044453163

The structure in **Figure S1.**
Energies: E= -3882.752658, solv = -0.075533, disp = -181.34 Z_0 = 695.17

Fe1	10.0668005743	48.8647390226	25.5989264873
N2	8.1082884519	48.5986992136	25.8932745833
N3	9.9631664120	50.3755866141	26.8593282508
N4	12.0613313194	49.2254745918	25.2854070520
N5	10.1316029088	47.2960565353	24.3347211439
C6	7.4390673720	47.4393351792	25.6799314192
O7	2.4580000422#	47.4399990091#	26.0699998977#
C8	8.8969647863	51.1483545347	27.0968892407
C9	12.8791918277	50.0576478108	25.9307705338
C10	11.1341440727	46.9310597631	23.4859779978
O11	8.8418527924	40.9520773118	21.9445179858
C12	6.0624418546	47.5470320223	26.1744140346

O13	3.2379634518	48.2542870664	24.1464929695
C14	9.2544522866	52.3776368654	27.8717746295
C15	14.1986723895	50.1670774985	25.2824374387
C16	10.7758276117	45.6955985475	22.7615648173
O17	7.2990000306#	41.4389989899#	23.5170000201#
C18	5.8628146677	48.8547498264	26.5507453643
C19	10.5714429365	52.2465026383	28.1692049923
C20	14.1278926942	49.3080120968	24.1982974618
C21	9.5430976371	45.3323748714	23.2537002625
C22	7.1952873289	49.4898779210	26.4043800917
C23	11.0379706547	50.8596289034	27.7114533543
C24	12.7925792657	48.6936826089	24.2167543521
C25	9.1586647686	46.3408542558	24.2350841563
C26	5.1507347794	46.3519674564	26.2317980208
C27	11.4485869640	53.0398433515	29.0712226652
C28	15.1943865502	48.9607338277	23.1971068455
C29	8.7305757094	44.1359570193	22.8414099973
C30	4.2257085114	46.2082569964	24.9973575193
C31	10.6800002095#	53.7659989444#	30.1639996530#
C32	16.5419997795#	48.6100011712#	23.8370000554#
C33	9.2412744825	42.8065713493	23.4574633762
C34	3.2618918439	47.3772332871	24.9719354743
C35	8.4832435884	41.6282257689	22.8816160177
C36	7.9451276219	46.3689962717	24.9331004945
C37	7.5582418450	50.7673895394	26.8179533807
C38	12.5466853485	50.6907867038	27.2612345093
C39	12.3543057895	47.6531169335	23.4067673672
C40	4.5840006015#	49.5429986614#	27.0259989337#
C41	8.2430004336#	53.4420002961#	28.2979997086#
C42	15.4039996458#	51.0260007769#	25.7099991889#
C43	11.5399995569#	44.9049995195#	21.7090011325#
C44	12.9969999455#	54.0449979434#	25.3990003933#
C45	12.0325033087	54.5131820393	26.4967042078
S46	12.6938053586	54.1574499833	28.1901103848
N47	13.2759896503	52.4723914488	22.4536067781
C48	13.3156345014	51.8653325144	21.1189233537
C49	11.8820863810	51.8476338433	20.5592390923
C50	13.9169270524	50.4429395643	21.0631702259
S51	15.5362918301	50.2955039974	21.9311154288
N52	11.3382309430	53.2031051590	20.5600179130
C53	9.8937626875	53.3422779461	20.6155419450
C54	9.2412516542	53.2613370029	22.0301080677
C55	9.2708905676	51.9276051565	22.7097609826
N56	8.5562698204	50.8378899257	22.2366322070
C57	9.9027241574	51.4551464102	23.8351590763
C58	8.7755052569	49.7814262912	23.0563787853
N59	9.5901496849	50.1225473714	24.0399962376
C60	16.9891349971#	48.9429349498#	31.0169701381#
C61	16.1408280871	48.1117862153	30.0318922541
C62	14.7388770543	48.6460588589	29.7842228315

C63	13.6995127052	48.3857726125	30.6932659293
C64	14.4468383239	49.3992073204	28.6332395314
C65	12.3948782756	48.8427738421	30.4806564413
C66	13.1529195279	49.8675632607	28.4048911102
C67	12.1528685192	49.5749094947	29.3226446336
O68	10.9210967775	50.0622658400	28.9689044393
H69	17.0250684908	49.4799534078	24.2857401456
H70	17.2227550196	48.2071133743	23.0810522048
H71	16.4013978688	47.8520501464	24.6148428860
H72	16.6705618886	48.0440735089	29.0734522863
H73	16.0669335878	47.0840667923	30.4102349736
H74	16.5087799684	49.0031696367	31.9998290158
H75	17.1303789958	49.9661535350	30.6516126051
H76	15.2410653894	49.6011690887	27.9243100642
H77	11.6013525804	48.6431326731	31.1940476371
H78	13.9108861936	47.8044823626	31.5873225095
H79	5.7516342927	45.4422904115	26.3327005425
H80	4.5441870748	46.4029298572	27.1435603840
H81	7.6770668254	44.2690524806	23.1077501463
H82	8.7563429660	44.0394839191	21.7486473218
H83	4.8009858303	46.2120965067	24.0692547630
H84	3.6742172883	45.2601016618	25.0538503062
H85	10.2976683925	42.6646565862	23.2190659122
H86	9.1267382687	42.8319647684	24.5456999645
H87	3.9053145560	48.8313100084	27.4975375147
H88	4.0516254885	49.9867471692	26.1783675578
H89	4.8088348123	50.3347641847	27.7475289125
H90	12.1116927206	45.5584975197	21.0405216031
H91	10.8508012239	44.3212686380	21.0936250277
H92	12.2511255382	44.2064567801	22.1676300045
H93	9.6194679859	54.3231498770	20.2053741296
H94	9.4419497424	52.5986846305	19.9452953549
H95	11.7726362944	53.7065818362	21.3323026085
H96	14.2282239572	52.5785367389	22.8000246902
H97	10.0054424173	53.0705091769	30.6758805698
H98	10.1037762399	54.6112480019	29.7864307170
H99	11.3771539326	54.1663488183	30.9072723550
H100	7.8682703647	53.2490294949	29.3090339625
H101	7.3860346439	53.4554304800	27.6182296351
H102	8.6836756177	54.4392424725	28.2805113085
H103	6.7715499748	51.4470031712	27.1218094822
H104	7.2910212094	45.5154341524	24.7956784997
H105	6.8642381022	40.6878425302	23.0686544936
H106	2.4924632448	46.6105906876	26.5738232201
H107	13.9199852455	52.5301545833	20.4897312221
H108	13.2246599392	49.7337716110	21.5299286320
H109	14.0600931898	50.1309444492	20.0232967348
H110	9.7390952629	53.9878801632	22.6825167035
H111	8.1973222345	53.5985505171	21.9442959302
H112	10.5575675993	51.9884163605	24.5058524976

H113	7.9628095047	50.8296846079	21.4183751200
H114	8.3234749962	48.8121402781	22.9204982517
H115	13.9865671579	54.4950251350	25.5302914054
H116	12.6279283216	54.3296194980	24.4075604968
H117	13.1167283761	52.9587392555	25.4115714862
H118	11.8722924876	55.5933730178	26.4351958334
H119	11.0552489439	54.0299452558	26.4018090620
H120	11.8899419636	51.4494351337	19.5345258486
H121	11.2943206854	51.1391495387	21.1708609674
H122	15.1161785349	51.7855479999	26.4416977245
H123	15.8249736273	51.5335646180	24.8364960041
H124	16.1958925553	50.4145389252	26.1528735305
H125	13.0563067282	47.2896230679	22.6627640735
H126	12.1275424164	52.3330189563	29.5600077297
H127	14.8713300185	48.0916462448	22.6195328426
H128	12.9953563232	51.6905515778	27.2755117004
H129	12.8143546350	51.8324437626	23.1007197544
H130	17.9766463920	48.4884699441	31.1494065651
H131	9.7478758419	48.4301849499	28.5157666402
N132	9.9234428004	47.5051695541	28.1138895904
H133	10.3549156178	46.8240389724	28.7371992669
O134	10.5016215912	47.5297571629	26.9374327432

The structure in **Figure S2.**
Energies: E= -3882.129389, solv = -0.076288, disp = -179.44 Z_0 = 687.16

Fe1	10.0518545532	48.9552610975	25.5444084278
N2	8.1244261266	48.6406118165	25.8775349577
N3	9.9602830789	50.4047201518	26.7805658597
N4	12.0430952914	49.2818918726	25.2364278282
N5	10.1417139030	47.3055788293	24.3528245115
C6	7.4693538085	47.4316093595	25.7261318264
O7	2.4580000305#	47.4399990117#	26.0699998972#
C8	8.8731961841	51.1856504318	27.0397954546
C9	12.8737452854	50.0980273284	25.9059221885
C10	11.1460624496	46.9498669902	23.4703802480
O11	8.8299750640	40.9911509328	21.9217871152
C12	6.1053256295	47.5510160912	26.2388299423
O13	3.3205391968	48.1811040057	24.1530796590
C14	9.2460841110	52.3763133241	27.8509122544
C15	14.1835783617	50.1871217330	25.2761626516
C16	10.7908750253	45.7179339136	22.7743625329
O17	7.2990000253#	41.4389989800#	23.5170000182#
C18	5.8875396398	48.8717438070	26.5586835383
C19	10.5551131696	52.2117001344	28.1748971571
C20	14.1132228393	49.3240468697	24.1820367845
C21	9.5585381266	45.3443418025	23.3013273283
C22	7.2059047458	49.5229567314	26.3627368771

C23	11.0194063854	50.8313651856	27.6864042646
C24	12.7798497228	48.7381470567	24.1768514769
C25	9.1859338568	46.3386830657	24.2758135076
C26	5.1894395552	46.3622132663	26.3447427461
C27	11.4336602856	53.0037294847	29.0811623449
C28	15.1923618790	48.9634426985	23.1961295162
C29	8.7427869602	44.1497400615	22.8843278389
C30	4.2699119754	46.1668685301	25.1120305494
C31	10.6800001964#	53.7659989562#	30.1639996875#
C32	16.5419997821#	48.6100011708#	23.8370000547#
C33	9.2446215449	42.8065359664	23.4771361421
C34	3.3091330140	47.3356973790	25.0116470699
C35	8.4794649660	41.6435937107	22.8785652316
C36	7.9705146419	46.3653457974	25.0103455374
C37	7.5571620141	50.8257154943	26.7528481569
C38	12.5352063030	50.6962692460	27.2478508946
C39	12.3458721380	47.6839619384	23.3762677799
C40	4.5840005876#	49.5429986604#	27.0259989383#
C41	8.2430004428#	53.4420002815#	28.2979997162#
C42	15.4039996363#	51.0260007543#	25.7099992055#
C43	11.5399995689#	44.9049995110#	21.7090011007#
C44	12.9969999477#	54.0449979395#	25.3990003779#
C45	12.0217747417	54.4789467361	26.5018384680
S46	12.6897831093	54.1099674082	28.1892791801
N47	13.2445251042	52.4216995521	22.4288532570
C48	13.3074098254	51.8224196001	21.0911231067
C49	11.8787460143	51.7773228943	20.5222153094
C50	13.9387604442	50.4137636872	21.0308570584
S51	15.5468509751	50.2863913666	21.9191496801
N52	11.3192724922	53.1253147937	20.5048953522
C53	9.8732984812	53.2471588912	20.5189392841
C54	9.1897032226	53.1889368115	21.9196817482
C55	9.2307917476	51.8746920019	22.6344760221
N56	8.5215974082	50.7655597205	22.1939771542
C57	9.8650086387	51.4453234876	23.7737095472
C58	8.7370688970	49.7367848420	23.0418130778
N59	9.5510676704	50.1190303478	24.0145153677
C60	16.9891350165#	48.9429349526#	31.0169701454#
C61	16.1408582387	48.1251006760	30.0178585480
C62	14.7314366172	48.6430726337	29.7650584537
C63	13.6771314380	48.3264813550	30.6397152608
C64	14.4407648890	49.4200191884	28.6287354657
C65	12.3641380532	48.7528586065	30.4100044402
C66	13.1387219090	49.8570753755	28.3824850527
C67	12.1233364426	49.5156630114	29.2693035544
O68	10.8907407690	49.9781978743	28.9005911098
H69	17.0339433639	49.4819636903	24.2721419832
H70	17.2143886852	48.1916168525	23.0823155371
H71	16.3985538355	47.8638711817	24.6257745811
H72	16.6742232079	48.0745778149	29.0601806480

H73	16.0745106928	47.0911809963	30.3806320482
H74	16.5104496910	48.9852213346	32.0016060195
H75	17.1281422808	49.9724580012	30.6688670674
H76	15.2433203537	49.6653256597	27.9433047207
H77	11.5603960961	48.5090911679	31.0976571664
H78	13.8830791928	47.7224069275	31.5200359586
H79	5.7851417509	45.4546632511	26.4875087659
H80	4.5772332074	46.4570069240	27.2494393127
H81	7.6904038340	44.2840261154	23.1555279073
H82	8.7636982714	44.0665550083	21.7902072568
H83	4.8535994357	46.1215026123	24.1903692268
H84	3.7144500556	45.2245917143	25.2109250573
H85	10.3001735552	42.6608934558	23.2371195307
H86	9.1290147705	42.8122168033	24.5656388027
H87	3.9277214589	48.8206876953	27.5119104918
H88	4.0412268743	49.9596604352	26.1718218713
H89	4.7932437191	50.3506537817	27.7346347680
H90	12.2403403510	45.5246485958	21.1399853588
H91	10.8317419649	44.4637814270	21.0021754461
H92	12.1136171854	44.0888652204	22.1635577518
H93	9.5941159805	54.2149469475	20.0817954246
H94	9.4478347752	52.4825305436	19.8552499622
H95	11.7294967458	53.6358345529	21.2859442590
H96	14.1926445479	52.5715331585	22.7703432800
H97	9.9881535676	53.0964782131	30.6876627897
H98	10.1255115240	54.6185069286	29.7710544632
H99	11.3887604101	54.1600287373	30.8991787996
H100	7.8803390115	53.2397956295	29.3111045842
H101	7.3792946470	53.4646762872	27.6279121941
H102	8.6877206581	54.4376269944	28.2851193793
H103	6.7644365893	51.4996898830	27.0512357901
H104	7.3362930831	45.4909290901	24.9163867335
H105	6.8606949795	40.7003673466	23.0516519828
H106	2.4784646426	46.6349817823	26.6129644325
H107	13.8999255141	52.5044921136	20.4694641545
H108	13.2547814928	49.6862417172	21.4814266445
H109	14.1018408251	50.1148205247	19.9900875302
H110	9.6593283670	53.9405279950	22.5643130114
H111	8.1412057159	53.5016939264	21.8053243872
H112	10.5165662268	51.9976623414	24.4306529024
H113	7.9284523943	50.7298013650	21.3757453498
H114	8.2921968173	48.7606002167	22.9388155426
H115	13.9758888552	54.5162799296	25.5354404138
H116	12.6206803733	54.3339143610	24.4112923664
H117	13.1416664491	52.9618859748	25.3987483013
H118	11.8382587154	55.5561095642	26.4534086383
H119	11.0553319122	53.9757299414	26.3995854971
H120	11.8975891294	51.3684145615	19.5020722701
H121	11.2968850170	51.0667957279	21.1386231876
H122	15.1283243486	51.7775592749	26.4547865330

H123	15.8250096176	51.5435463112	24.8421153128
H124	16.1924043356	50.4003234650	26.1387367993
H125	13.0518046589	47.3183180497	22.6372218606
H126	12.1076907895	52.2979200962	29.5783024968
H127	14.8696259745	48.0924083778	22.6213486987
H128	12.9718589409	51.7002203710	27.2863629767
H129	12.8236373754	51.7545070147	23.0763264923
H130	17.9771240610	48.4867589021	31.1385344995
N131	9.9753910719	47.3677820491	27.8871258885
H132	10.5349013209	46.6975748750	28.4718007201
O133	10.6819087539	47.6579565388	26.9337821319

The structure in **Figure 4.**
Energies: E= -3881.553136, solv = -0.072550, disp = -178.15 Z_0 = 680.58

Fe1	10.0726834889	48.8836214304	25.5769555463
N2	8.1089969499	48.6151438553	25.8681220752
N3	9.9631994562	50.4394575427	26.7626003630
N4	12.0590822411	49.2549557202	25.2465859769
N5	10.1344753305	47.2942483508	24.3380508481
C6	7.4464185096	47.4488860589	25.6816519539
O7	2.4580000499#	47.4399990063#	26.0699999333#
C8	8.8968628977	51.1862282943	27.0356618810
C9	12.8781344132	50.0801835527	25.9000308933
C10	11.1374249388	46.9336791406	23.4813933739
O11	8.8529179245	40.9373958689	21.9600743250
C12	6.0745998525	47.5570282509	26.1903746159
O13	3.2764274173	48.2141182154	24.1462201926
C14	9.2520918900	52.3836395717	27.8613313123
C15	14.2010607438	50.1753986123	25.2625182429
C16	10.7780660917	45.6966116046	22.7651061422
O17	7.2990001082#	41.4389991241#	23.5169999937#
C18	5.8683248537	48.8689630607	26.5434427279
C19	10.5631761202	52.2304494469	28.1747811870
C20	14.1312416502	49.3173366354	24.1768793065
C21	9.5474872082	45.3287614488	23.2652128656
C22	7.1958475427	49.5141009555	26.3731379443
C23	11.0302464614	50.8596202019	27.6657477696
C24	12.7925581452	48.7107884840	24.1865629516
C25	9.1623202370	46.3361499298	24.2446094688
C26	5.1676522993	46.3604835063	26.2802223130
C27	11.4424377797	53.0120745674	29.0848170362
C28	15.2015019581	48.9611353977	23.1841080546
C29	8.7335280229	44.1319315317	22.8550506315
C30	4.2415681891	46.1816665148	25.0506819333
C31	10.6800001749#	53.7659988481#	30.1639996024#
C32	16.5419998189#	48.6100011123#	23.8370000409#
C33	9.2472627343	42.8004032895	23.4637367995

C34	3.2808885378	47.3524190171	24.9882251801
C35	8.4881993899	41.6211108086	22.8889487011
C36	7.9520262798	46.3670209614	24.9497120047
C37	7.5533518434	50.7977916210	26.7613679797
C38	12.5411833643	50.7190838165	27.2233882312
C39	12.3534913247	47.6610258504	23.3886430296
C40	4.5840004466#	49.5429988461#	27.0259989286#
C41	8.2430002602#	53.4420006433#	28.2979997519#
C42	15.4039997807#	51.0260009164#	25.7099990708#
C43	11.5399996560#	44.9049994118#	21.7090006845#
C44	12.9969999734#	54.0449979877#	25.3990002887#
C45	12.0432012019	54.4961762743	26.5145559771
S46	12.7140035229	54.1020861251	28.1972557918
N47	13.2306644480	52.4177637062	22.4237481718
C48	13.3119040695	51.8263284963	21.0839978513
C49	11.8917629106	51.7868457855	20.4935643248
C50	13.9428744568	50.4170974201	21.0243728827
S51	15.5513918731	50.2897597995	21.9154116714
N52	11.3311177266	53.1345353902	20.4819239490
C53	9.8839426034	53.2548586499	20.4735859428
C54	9.1790238498	53.1893359618	21.8629518500
C55	9.2207537714	51.8727124727	22.5739830826
N56	8.5216048699	50.7622546985	22.1253398513
C57	9.8595226309	51.4348595886	23.7091223800
C58	8.7558123637	49.7286374844	22.9692162592
N59	9.5663527884	50.1030926716	23.9437999991
C60	16.9891349711#	48.9429349907#	31.0169700324#
C61	16.1281459670	48.1447061299	30.0145319593
C62	14.7251897567	48.6806374062	29.7631510142
C63	13.6626176042	48.3610152180	30.6264835434
C64	14.4470475464	49.4659175462	28.6300402492
C65	12.3508611621	48.7817446153	30.3838569418
C66	13.1453548160	49.8960331772	28.3707852859
C67	12.1203854123	49.5365680099	29.2381585695
O68	10.8863462856	49.9848985277	28.8470842936
H69	17.0259068221	49.4818067837	24.2810702837
H70	17.2265387278	48.1974741867	23.0897913652
H71	16.3927534677	47.8599412501	24.6208007440
H72	16.6603461604	48.0919399611	29.0561749825
H73	16.0469039108	47.1095740476	30.3703772375
H74	16.5123480575	48.9862431925	32.0025084451
H75	17.1434543301	49.9727112171	30.6757468312
H76	15.2582727229	49.7183604151	27.9578369482
H77	11.5371788809	48.5180596497	31.0510861803
H78	13.8598689224	47.7511345155	31.5046461572
H79	5.7715007839	45.4557990032	26.4055441023
H80	4.5616489972	46.4350035823	27.1905893499
H81	7.6816544182	44.2647214456	23.1275382494
H82	8.7532502077	44.0397061945	21.7617883429
H83	4.8166622894	46.1559418764	24.1227503036

H84	3.6892017716	45.2364246401	25.1366103709
H85	10.3023687855	42.6586386383	23.2198589174
H86	9.1383294500	42.8215969239	24.5527795402
H87	3.9201711004	48.8232463283	27.5058072955
H88	4.0394991284	49.9759989998	26.1805963410
H89	4.8041997437	50.3401833409	27.7429639861
H90	12.1185532121	45.5577537169	21.0461542555
H91	10.8472474554	44.3304707432	21.0889889154
H92	12.2431325847	44.1984526015	22.1671233739
H93	9.6114013153	54.2236936842	20.0343503648
H94	9.4706838696	52.4916253309	19.8008201543
H95	11.7272185022	53.6380148368	21.2745465002
H96	14.1728751770	52.5424388013	22.7905316933
H97	9.9848398136	53.0916319315	30.6765587514
H98	10.1272491170	54.6202840921	29.7719067942
H99	11.3800265764	54.1569904753	30.9092042223
H100	7.8685512293	53.2415474399	29.3075855677
H101	7.3852009137	53.4628774773	27.6195246524
H102	8.6828553914	54.4398369326	28.2888676763
H103	6.7656071667	51.4720409683	27.0738627981
H104	7.3034693243	45.5064474173	24.8343482981
H105	6.8663954065	40.6843333481	23.0724891544
H106	2.4791440615	46.6206846902	26.5910312233
H107	13.9152610993	52.5106443746	20.4752820325
H108	13.2576972995	49.6879505533	21.4705666961
H109	14.1101991764	50.1199415027	19.9837857853
H110	9.6340904813	53.9431521860	22.5157494133
H111	8.1307344327	53.4966982258	21.7323325856
H112	10.5078588140	51.9917169206	24.3669579834
H113	7.9267584559	50.7281934773	21.3085177701
H114	8.3167017666	48.7502870569	22.8553193478
H115	13.9897068761	54.4877763016	25.5305212070
H116	12.6194302720	54.3536955837	24.4179121923
H117	13.1109810232	52.9583705187	25.3865859232
H118	11.8877540618	55.5779800744	26.4741696687
H119	11.0631132011	54.0193038213	26.4162239513
H120	11.9272968783	51.3890331713	19.4694064930
H121	11.3011164093	51.0692679399	21.0928886067
H122	15.1136003796	51.7708834494	26.4551402236
H123	15.8291921144	51.5501894155	24.8479260103
H124	16.1936680468	50.4054323299	26.1434434684
H125	13.0538011568	47.2911571521	22.6463607731
H126	12.1081314848	52.2983504611	29.5822161293
H127	14.8787907215	48.0905552938	22.6083149330
H128	12.9793743098	51.7222732473	27.2352816997
H129	12.7705962108	51.7605473958	23.0541912398
H130	17.9704314337	48.4714895305	31.1349300320
O131	10.0259927992	46.8100760724	27.4835122925
N132	10.4585775981	47.7574159764	26.9586092421

The structure in **Figure S3.**

Energies: E= -3768.276699, solv = -0.157619, disp = -173.62 Z_0 = 668.19

Fe1	0.9915120936	-6.6392464264	1.5642509159
C2	0.8693783577	-3.6390648777	3.2411224752
C3	-0.2124479416	-8.2126259355	4.3236975359
C4	0.2469631369	-9.3861961223	-0.3621261738
C5	2.6792884149	-5.2499270561	-0.9460107568
N6	0.5457600169	-6.0573232482	3.4358951218
C7	0.8035974768	-4.8456053321	4.0220232396
C8	0.8727024629	-4.9561728173	5.4414743588
C9	0.4904568677	-6.2556998670	5.7361376761
C10	0.2861272703	-6.9272982406	4.4887267583
C11	0.2743430334#	-6.7888710128#	7.1082149867#
C12	1.4506953723	-3.9806636144	6.4395895489
C13	2.9289279163	-4.2996087218	6.7523380858
C14	3.8297630278#	-3.8698639912#	5.6169419994#
O15	4.9375099626	-4.6522665134	5.4977294828
O16	3.6373257813	-2.9221069739	4.8862347834
N17	0.1571680302	-8.4634832927	1.8915204577
C18	-0.3668506068	-8.8775160267	3.1082469340
C19	-1.0505611985	-10.1162122890	2.9582903378
C20	-0.8393217641	-10.5338671912	1.6407219100
C21	-0.0787428970	-9.4617135897	0.9889639406
C22	-1.7618710794#	-10.8824639052#	4.0177929796#
C23	-1.3842925432	-11.7616346163	1.1227675538
C24	-2.8547534711#	-11.9954430134#	1.1219388878#
N25	1.3033698319	-7.1617625943	-0.3059951243
C26	0.9648770048	-8.3309642238	-0.9309787464
C27	1.5120494230	-8.3742209377	-2.2749600635
C28	2.2310533226	-7.2081900214	-2.4525291720
C29	2.1017555103	-6.4698594837	-1.2016773416
C30	1.2334229274#	-9.5722279931#	-3.1310109934#
C31	2.9276984311	-6.5847423793	-3.6511590670
C32	2.8164840612#	-7.3065219915#	-4.9860738194#
N33	1.7175467910	-4.8183122762	1.2676066704
C34	2.4997956273	-4.4736444518	0.1991159709
C35	2.9961652845	-3.1389878891	0.3353519894
C36	2.4061159665	-2.6143467461	1.4747308927
C37	1.6133022854	-3.6749095433	2.0284049600
C38	3.9804168155	-2.4833191884	-0.5869579250
C39	2.7016482000	-1.2569417330	2.0662003846
C40	2.1716179553	-0.0707661191	1.2422129972
C41	0.6707260323#	-0.0054000251#	1.0951810138#
O42	0.3139387143	0.7990059603	0.0884431724
O43	-0.1636340867	-0.5581504869	1.7983075468
H44	-0.5536696023	-8.7249874235	5.2151202702

H45	-0.0135727070	-10.2219956348	-0.9978917759
H46	3.3131799141	-4.8234670570	-1.7070431638
H47	1.1313113376	-6.5920929500	7.7619059415
H48	0.1080176958	-7.8694926405	7.1206530067
H49	-0.5985123162	-6.3162913872	7.5766890362
H50	0.8744683435	-4.0069906986	7.3704570418
H51	1.4284326921	-2.9558299170	6.0684806403
H52	3.0954085554	-5.3546756364	6.9846820142
H53	3.2512345948	-3.7275762582	7.6333332906
H54	-1.3345227439	-10.6985199569	5.0078757615
H55	-1.7121849935	-11.9618803191	3.8496070016
H56	-2.8207699638	-10.6000003312	4.0793662721
H57	1.6160936194	-10.4881854574	-2.6645045239
H58	1.6914574236	-9.4988918910	-4.1166185982
H59	0.1557098424	-9.7085562529	-3.2847039973
H60	1.7684926692	-7.4880887735	-5.2526120337
H61	3.3372317453	-8.2689565135	-4.9852772768
H62	4.5043667859	-1.6637985344	-0.0857661800
H63	3.4934373378	-2.0617256491	-1.4765099908
H64	4.7390177977	-3.1927418542	-0.9330437298
H65	3.7904279967	-1.1405796730	2.1275204072
H66	2.3504820961	-1.1945796134	3.0980848808
H67	2.6062408122	-0.0542021013	0.2383467576
H68	2.4789087600	0.8737300144	1.7125654830
C69	-3.2559239845#	-3.5334329891#	7.9400739711#
C70	-2.7672530123	-3.9092332625	6.5415757512
C71	-3.0725045904	-2.8377788106	5.4771073811
C72	-2.1226136237	-2.9048524218	4.2688755929
C73	-0.7576600179	-2.3016894943	4.6453437523
N74	0.2902560868	-2.5040013814	3.6363787375
H75	-1.6848861249	-4.0932358008	6.5846829209
H76	-3.2160704544	-4.8618914017	6.2275178965
H77	-4.1106913018	-2.9391883470	5.1403497147
H78	-3.0027739782	-1.8371189992	5.9275363521
H79	-1.9908118617	-3.9509788929	3.9580559284
H80	-2.5314417002	-2.3624236032	3.4088355222
H81	-0.8558666455	-1.2171449905	4.7472188644
H82	-0.4413514109	-2.6780013136	5.6134240836
C83	2.0373817091#	-12.3268851563#	0.0888090568#
C84	1.1656404047	-12.9478229856	1.1519970777
S85	-0.5552021583	-13.1349143445	0.5817593611
H86	1.4697939239	-13.9736122084	1.3854352817
H87	1.1434120156	-12.3693692694	2.0798852524
C88	7.0061217697	-7.4386671955	-2.7777175526
C89	6.9798086830	-7.2310039396	-1.2662670689
O90	6.9529526160	-6.1334408492	-0.7293659547
C91	5.6025414033	-7.6745577808	-3.3672234894
S92	4.6985631498	-6.0821079228	-3.3579818213
H93	5.6939473909	-8.0461341466	-4.3917339933
H94	5.0535817789	-8.4154044244	-2.7748754687

N95	6.9436695383	-8.4098099702	-0.5450816776
C96	7.1737737442#	-8.4196829223#	0.8846719177#
C97	6.0386381484	-9.0904636438	1.7070782157
C98	4.8216866873	-8.2520124579	1.9593821563
N99	4.7950663175	-7.2553758403	2.9287765312
C100	3.5667234028	-8.2308651694	1.4093486511
C101	3.5816367295	-6.6740706380	2.9353880575
N102	2.8064760117	-7.2456058764	2.0222260842
H103	7.1651299526	-9.2607501042	-1.0457778588
H104	7.3126214737	-7.3781516229	1.1850113565
H105	5.7290282291	-10.0140676632	1.2061298962
H106	6.4591743548	-9.3990035215	2.6738905032
H107	5.5508861096	-7.0091388193	3.5549800670
H108	3.1670819582	-8.8383267760	0.6151155961
H109	3.3028832258	-5.8627177788	3.5856572137
H110	5.5170761980	-4.1929679364	4.8574806836
H111	-0.6617406285	0.8557353012	0.0755630511
H112	3.2611561969	-6.6894546213	-5.7706006097
H113	2.4702455838	-5.5956554175	-3.7818076051
H114	3.0841978683	-12.3520996267	0.4145162748
H115	1.7672111964	-11.2881565976	-0.0967538009
H116	1.9785080859	-12.8823018816	-0.8528983267
H117	7.4645444943	-6.5538601100	-3.2281811230
H118	7.6386315506	-8.2985101962	-3.0327729316
H119	-4.3356548231	-3.3441438959	7.9481916427
H120	-2.7604087971	-2.6237405135	8.2991840722
H121	-3.0576850506	-4.3315934992	8.6642063322
H122	-3.1318325108	-12.9634851028	0.6965924560
H123	-3.3658473890	-11.1931355663	0.5758958046
H124	-3.2398538098	-11.9590139839	2.1489185984
H125	8.1048022277	-8.9572698036	1.1035703075
H126	0.2918493746	-1.7508510778	2.9370222829
H127	-1.3665993289	-7.3755930912	-0.0784322316
N128	-1.8581705519	-6.5411233292	0.2443373926
H129	-1.8856714936	-5.9022076601	-0.5522236762
O130	-0.8986318026	-5.8930872617	1.1237095086
H131	-1.3486202566	-5.8598887017	1.9861293681

The structure of NH_2O bound to cyt-P460
Energies: E= -3767.659741, solv = -0.152182, disp = -171.86 Z_0 = 678.10

Fe1	0.8327465268	-6.5806657123	1.5493854451
C2	0.6634935387	-3.6101422898	3.2356685360
C3	-0.2150548340	-8.1986872932	4.3315339819
C4	0.2064639673	-9.3765322504	-0.3661933641
C5	2.3573410478	-5.1066838058	-1.0427036687
N6	0.4851564120	-6.0383755754	3.4336154852
C7	0.6741363021	-4.8208857723	4.0058931912

C8	0.7881031664	-4.9150609649	5.4307436325
C9	0.4586656391	-6.2204491395	5.7420724291
C10	0.2646675893	-6.9099320957	4.4905633898
C11	0.2743430664#	-6.7888710092#	7.1082149588#
C12	1.3719671787	-3.8763871167	6.3603531332
C13	2.8435559589	-4.1431927042	6.7358350983
C14	3.8297630241#	-3.8698639846#	5.6169420328#
O15	5.0925597736	-3.9059253187	6.0811047622
O16	3.5663162198	-3.6443381880	4.4494881565
N17	0.1237375883	-8.4394238167	1.8912441987
C18	-0.3873024153	-8.8608930898	3.1113843244
C19	-1.0629893081	-10.1007509383	2.9620467912
C20	-0.8538632439	-10.5196442587	1.6426861368
C21	-0.1085136361	-9.4401815100	0.9874043248
C22	-1.7618710778#	-10.8824639069#	4.0177929774#
C23	-1.3841472443	-11.7600685831	1.1434419039
C24	-2.8547534606#	-11.9954430122#	1.1219388859#
N25	1.1040253861	-7.0898517920	-0.3648514734
C26	0.8652007139	-8.3009360253	-0.9620028233
C27	1.4304167533	-8.3400869817	-2.2979414828
C28	2.0762220131	-7.1325594511	-2.4949635174
C29	1.8663878426	-6.3675850398	-1.2744700551
C30	1.2334229224#	-9.5722280124#	-3.1310110036#
C31	2.7790853144	-6.5070270780	-3.6911720301
C32	2.8164840778#	-7.3065219904#	-4.9860737889#
N33	1.4199244314	-4.7149973668	1.1908383620
C34	2.1854751980	-4.3479917564	0.1150284667
C35	2.7401252979	-3.0459837024	0.3052931025
C36	2.2139402828	-2.5666565006	1.4965725747
C37	1.3810940401	-3.6107228907	2.0043875720
C38	3.7401481233	-2.3884203123	-0.5986822416
C39	2.5924764810	-1.2747779518	2.1741021003
C40	2.1459540712	-0.0260314721	1.3994296790
C41	0.6707260444#	-0.0054000452#	1.0951810226#
O42	0.4001196033	0.7392919198	0.0168461366
O43	-0.2204558187	-0.5417063581	1.7389425577
H44	-0.5303561684	-8.7202205479	5.2285235264
H45	-0.0250953887	-10.2351597511	-0.9829189331
H46	2.9539633773	-4.6473171717	-1.8133543272
H47	0.4896299319	-6.0480153402	7.8817411861
H48	0.9191361677	-7.6567899837	7.2941883493
H49	-0.7610566528	-7.1211571335	7.2577410955
H50	0.7925665457	-3.8269244453	7.2894440520
H51	1.3288016008	-2.8830853367	5.9107000699
H52	2.9958242850	-5.1767077481	7.0710441372
H53	3.1333794414	-3.5124038117	7.5843108763
H54	-1.3158578191	-10.7225839658	5.0044058690
H55	-1.7266783405	-11.9582698110	3.8256152973
H56	-2.8173362655	-10.5931865064	4.1070481985
H57	1.6653657575	-10.4532434842	-2.6411814634

H58	1.6932379664	-9.4932986176	-4.1151650850
H59	0.1667528203	-9.7756267370	-3.2874740058
H60	1.8067303981	-7.5861577816	-5.3079973218
H61	3.4077361811	-8.2225924109	-4.8905848053
H62	4.3151814682	-1.6281740707	-0.0614907822
H63	3.2608037938	-1.8914023268	-1.4525365737
H64	4.4545555290	-3.1156609104	-0.9977400326
H65	3.6840635902	-1.2336600467	2.2726468463
H66	2.2038273297	-1.2466423295	3.1941489469
H67	2.6872559051	0.0789497505	0.4556919967
H68	2.3663728663	0.8820264307	1.9777288029
C69	-3.2559239751#	-3.5334329856#	7.9400739519#
C70	-3.0744893311	-3.8924660346	6.4624273861
C71	-3.2914223406	-2.6980685519	5.5041771679
C72	-2.3616834032	-2.7273549299	4.2768112118
C73	-0.9418770866	-2.3191272010	4.7048491485
N74	0.0711141468	-2.4912197259	3.6541159083
H75	-2.0663285447	-4.3067534485	6.3205954700
H76	-3.7622176507	-4.7028749691	6.1881177343
H77	-4.3355780993	-2.6716933654	5.1730028083
H78	-3.1302356848	-1.7542970980	6.0448983229
H79	-2.3357527967	-3.7368550097	3.8446374884
H80	-2.7176645903	-2.0489165875	3.4927898381
H81	-0.9272212512	-1.2570291110	4.9690971179
H82	-0.6643833130	-2.8727851988	5.5952964578
C83	2.0373817063#	-12.3268851595#	0.0888090572#
C84	1.1796632147	-12.9129942950	1.1829434784
S85	-0.5414525007	-13.1399149366	0.6305379982
H86	1.4989408960	-13.9237006095	1.4577316442
H87	1.1552371242	-12.2963636030	2.0859709814
C88	6.8923719423	-6.8234938830	-2.5511133278
C89	6.8061860060	-6.9031267684	-1.0297591189
O90	6.5002398700	-5.9654623560	-0.2970195249
C91	5.5834903989	-7.2624598813	-3.2491657023
S92	4.4784863942	-5.8074456622	-3.3471983027
H93	5.8068940543	-7.6259842133	-4.2558740722
H94	5.0951130253	-8.0690351452	-2.6912144038
N95	7.0782809600	-8.1521666674	-0.5357121405
C96	7.1737736727#	-8.4196828939#	0.8846719061#
C97	5.8876016661	-8.9914759853	1.5281516675
C98	4.7284451715	-8.0472503223	1.6666538445
N99	4.8707487681	-6.7531107184	2.1493027542
C100	3.3768639765	-8.2205222056	1.5235177606
C101	3.6567665578	-6.1855954300	2.2566155616
N102	2.7180118015	-7.0550363715	1.8905771927
H103	7.4299958391	-8.8549621804	-1.1707138643
H104	7.4850656179	-7.4961540370	1.3823826608
H105	5.5559568280	-9.8646545214	0.9558686517
H106	6.1626874657	-9.3635868679	2.5260057221
H107	5.7382652573	-6.2300694869	2.1365692189

H108	2.8321096948	-9.0889334651	1.1920131914
H109	3.4992531481	-5.1733381157	2.5932222704
H110	5.6891195928	-3.6933155735	5.3381689679
H111	-0.5707947753	0.7738144600	-0.0910309282
H112	3.2651890366	-6.6997305990	-5.7760711344
H113	2.2423580524	-5.5732931283	-3.9040518405
H114	3.0858476435	-12.3135148607	0.4109002234
H115	1.7449051143	-11.3046248460	-0.1485724165
H116	1.9882008294	-12.9289876021	-0.8243016289
H117	7.1519027620	-5.7975201580	-2.8270176644
H118	7.7079035616	-7.4647787729	-2.9068912885
H119	-4.2561200874	-3.1283607563	8.1317214642
H120	-2.5298247915	-2.7733934847	8.2533179187
H121	-3.1264244623	-4.4093856497	8.5852118812
H122	-3.1260682173	-13.0358926823	0.9280876374
H123	-3.3093136810	-11.3571927340	0.3504670171
H124	-3.3094439744	-11.6819946512	2.0683572123
H125	7.9751545415	-9.1480223311	1.0425749420
H126	0.0596486341	-1.7287487656	2.9632129818
H127	-0.9638484344	-6.5364861346	-0.6979884716
N128	-1.6024760810	-6.2353149297	0.0431967008
H129	-2.4868289160	-5.7724834895	-0.1575819066
O130	-1.0703320641	-5.9584835923	1.1831394871

The structure of NHO bound to cyt-P460
Energies: E= -3767.038969, solv = -0.147965, disp = -169.29 Z_0 = 670.04

Fe1	0.8327465268	-6.5806657123	1.5493854451
C2	0.6634935387	-3.6101422898	3.2356685360
C3	-0.2150548340	-8.1986872932	4.3315339819
C4	0.2064639673	-9.3765322504	-0.3661933641
C5	2.3573410478	-5.1066838058	-1.0427036687
N6	0.4851564120	-6.0383755754	3.4336154852
C7	0.6741363021	-4.8208857723	4.0058931912
C8	0.7881031664	-4.9150609649	5.4307436325
C9	0.4586656391	-6.2204491395	5.7420724291
C10	0.2646675893	-6.9099320957	4.4905633898
C11	0.2743430664#	-6.7888710092#	7.1082149588#
C12	1.3719671787	-3.8763871167	6.3603531332
C13	2.8435559589	-4.1431927042	6.7358350983
C14	3.8297630241#	-3.8698639846#	5.6169420328#
O15	5.0925597736	-3.9059253187	6.0811047622
O16	3.5663162198	-3.6443381880	4.4494881565
N17	0.1237375883	-8.4394238167	1.8912441987
C18	-0.3873024153	-8.8608930898	3.1113843244
C19	-1.0629893081	-10.1007509383	2.9620467912
C20	-0.8538632439	-10.5196442587	1.6426861368

C21	-0.1085136361	-9.4401815100	0.9874043248
C22	-1.7618710778#	-10.8824639069#	4.0177929774#
C23	-1.3841472443	-11.7600685831	1.1434419039
C24	-2.8547534606#	-11.9954430122#	1.1219388859#
N25	1.1040253861	-7.0898517920	-0.3648514734
C26	0.8652007139	-8.3009360253	-0.9620028233
C27	1.4304167533	-8.3400869817	-2.2979414828
C28	2.0762220131	-7.1325594511	-2.4949635174
C29	1.8663878426	-6.3675850398	-1.2744700551
C30	1.2334229224#	-9.5722280124#	-3.1310110036#
C31	2.7790853144	-6.5070270780	-3.6911720301
C32	2.8164840778#	-7.3065219904#	-4.9860737889#
N33	1.4199244314	-4.7149973668	1.1908383620
C34	2.1854751980	-4.3479917564	0.1150284667
C35	2.7401252979	-3.0459837024	0.3052931025
C36	2.2139402828	-2.5666565006	1.4965725747
C37	1.3810940401	-3.6107228907	2.0043875720
C38	3.7401481233	-2.3884203123	-0.5986822416
C39	2.5924764810	-1.2747779518	2.1741021003
C40	2.1459540712	-0.0260314721	1.3994296790
C41	0.6707260444#	-0.0054000452#	1.0951810226#
O42	0.4001196033	0.7392919198	0.0168461366
O43	-0.2204558187	-0.5417063581	1.7389425577
H44	-0.5303561684	-8.7202205479	5.2285235264
H45	-0.0250953887	-10.2351597511	-0.9829189331
H46	2.9539633773	-4.6473171717	-1.8133543272
H47	0.4896299319	-6.0480153402	7.8817411861
H48	0.9191361677	-7.6567899837	7.2941883493
H49	-0.7610566528	-7.1211571335	7.2577410955
H50	0.7925665457	-3.8269244453	7.2894440520
H51	1.3288016008	-2.8830853367	5.9107000699
H52	2.9958242850	-5.1767077481	7.0710441372
H53	3.1333794414	-3.5124038117	7.5843108763
H54	-1.3158578191	-10.7225839658	5.0044058690
H55	-1.7266783405	-11.9582698110	3.8256152973
H56	-2.8173362655	-10.5931865064	4.1070481985
H57	1.6653657575	-10.4532434842	-2.6411814634
H58	1.6932379664	-9.4932986176	-4.1151650850
H59	0.1667528203	-9.7756267370	-3.2874740058
H60	1.8067303981	-7.5861577816	-5.3079973218
H61	3.4077361811	-8.2225924109	-4.8905848053
H62	4.3151814682	-1.6281740707	-0.0614907822
H63	3.2608037938	-1.8914023268	-1.4525365737
H64	4.4545555290	-3.1156609104	-0.9977400326
H65	3.6840635902	-1.2336600467	2.2726468463
H66	2.2038273297	-1.2466423295	3.1941489469
H67	2.6872559051	0.0789497505	0.4556919967
H68	2.3663728663	0.8820264307	1.9777288029
C69	-3.2559239751#	-3.5334329856#	7.9400739519#
C70	-3.0744893311	-3.8924660346	6.4624273861

C71	-3.2914223406	-2.6980685519	5.5041771679
C72	-2.3616834032	-2.7273549299	4.2768112118
C73	-0.9418770866	-2.3191272010	4.7048491485
N74	0.0711141468	-2.4912197259	3.6541159083
H75	-2.0663285447	-4.3067534485	6.3205954700
H76	-3.7622176507	-4.7028749691	6.1881177343
H77	-4.3355780993	-2.6716933654	5.1730028083
H78	-3.1302356848	-1.7542970980	6.0448983229
H79	-2.3357527967	-3.7368550097	3.8446374884
H80	-2.7176645903	-2.0489165875	3.4927898381
H81	-0.9272212512	-1.2570291110	4.9690971179
H82	-0.6643833130	-2.8727851988	5.5952964578
C83	2.0373817063#	-12.3268851595#	0.0888090572#
C84	1.1796632147	-12.9129942950	1.1829434784
S85	-0.5414525007	-13.1399149366	0.6305379982
H86	1.4989408960	-13.9237006095	1.4577316442
H87	1.1552371242	-12.2963636030	2.0859709814
C88	6.8923719423	-6.8234938830	-2.5511133278
C89	6.8061860060	-6.9031267684	-1.0297591189
O90	6.5002398700	-5.9654623560	-0.2970195249
C91	5.5834903989	-7.2624598813	-3.2491657023
S92	4.4784863942	-5.8074456622	-3.3471983027
H93	5.8068940543	-7.6259842133	-4.2558740722
H94	5.0951130253	-8.0690351452	-2.6912144038
N95	7.0782809600	-8.1521666674	-0.5357121405
C96	7.1737736727#	-8.4196828939#	0.8846719061#
C97	5.8876016661	-8.9914759853	1.5281516675
C98	4.7284451715	-8.0472503223	1.6666538445
N99	4.8707487681	-6.7531107184	2.1493027542
C100	3.3768639765	-8.2205222056	1.5235177606
C101	3.6567665578	-6.1855954300	2.2566155616
N102	2.7180118015	-7.0550363715	1.8905771927
H103	7.4299958391	-8.8549621804	-1.1707138643
H104	7.4850656179	-7.4961540370	1.3823826608
H105	5.5559568280	-9.8646545214	0.9558686517
H106	6.1626874657	-9.3635868679	2.5260057221
H107	5.7382652573	-6.2300694869	2.1365692189
H108	2.8321096948	-9.0889334651	1.1920131914
H109	3.4992531481	-5.1733381157	2.5932222704
H110	5.6891195928	-3.6933155735	5.3381689679
H111	-0.5707947753	0.7738144600	-0.0910309282
H112	3.2651890366	-6.6997305990	-5.7760711344
H113	2.2423580524	-5.5732931283	-3.9040518405
H114	3.0858476435	-12.3135148607	0.4109002234
H115	1.7449051143	-11.3046248460	-0.1485724165
H116	1.9882008294	-12.9289876021	-0.8243016289
H117	7.1519027620	-5.7975201580	-2.8270176644
H118	7.7079035616	-7.4647787729	-2.9068912885
H119	-4.2561200874	-3.1283607563	8.1317214642
H120	-2.5298247915	-2.7733934847	8.2533179187

H121	-3.1264244623	-4.4093856497	8.5852118812
H122	-3.1260682173	-13.0358926823	0.9280876374
H123	-3.3093136810	-11.3571927340	0.3504670171
H124	-3.3094439744	-11.6819946512	2.0683572123
H125	7.9751545415	-9.1480223311	1.0425749420
H126	0.0596486341	-1.7287487656	2.9632129818
H127	-0.9638484344	-6.5364861346	-0.6979884716
N128	-1.6024760810	-6.2353149297	0.0431967008
H129	-2.4868289160	-5.7724834895	-0.1575819066
O130	-1.0703320641	-5.9584835923	1.1831394871

The structure of NO bound to cyt-P460

Energies: E= -3766.461026, solv = -0.145580, disp = -168.40 Z_0 = 663.74

Fe1	0.8327465268	-6.5806657123	1.5493854451
C2	0.6634935387	-3.6101422898	3.2356685360
C3	-0.2150548340	-8.1986872932	4.3315339819
C4	0.2064639673	-9.3765322504	-0.3661933641
C5	2.3573410478	-5.1066838058	-1.0427036687
N6	0.4851564120	-6.0383755754	3.4336154852
C7	0.6741363021	-4.8208857723	4.0058931912
C8	0.7881031664	-4.9150609649	5.4307436325
C9	0.4586656391	-6.2204491395	5.7420724291
C10	0.2646675893	-6.9099320957	4.4905633898
C11	0.2743430664#	-6.7888710092#	7.1082149588#
C12	1.3719671787	-3.8763871167	6.3603531332
C13	2.8435559589	-4.1431927042	6.7358350983
C14	3.8297630241#	-3.8698639846#	5.6169420328#
O15	5.0925597736	-3.9059253187	6.0811047622
O16	3.5663162198	-3.6443381880	4.4494881565
N17	0.1237375883	-8.4394238167	1.8912441987
C18	-0.3873024153	-8.8608930898	3.1113843244
C19	-1.0629893081	-10.1007509383	2.9620467912
C20	-0.8538632439	-10.5196442587	1.6426861368
C21	-0.1085136361	-9.4401815100	0.9874043248
C22	-1.7618710778#	-10.8824639069#	4.0177929774#
C23	-1.3841472443	-11.7600685831	1.1434419039
C24	-2.8547534606#	-11.9954430122#	1.1219388859#
N25	1.1040253861	-7.0898517920	-0.3648514734
C26	0.8652007139	-8.3009360253	-0.9620028233
C27	1.4304167533	-8.3400869817	-2.2979414828
C28	2.0762220131	-7.1325594511	-2.4949635174
C29	1.8663878426	-6.3675850398	-1.2744700551
C30	1.2334229224#	-9.5722280124#	-3.1310110036#
C31	2.7790853144	-6.5070270780	-3.6911720301
C32	2.8164840778#	-7.3065219904#	-4.9860737889#
N33	1.4199244314	-4.7149973668	1.1908383620
C34	2.1854751980	-4.3479917564	0.1150284667

C35	2.7401252979	-3.0459837024	0.3052931025
C36	2.2139402828	-2.5666565006	1.4965725747
C37	1.3810940401	-3.6107228907	2.0043875720
C38	3.7401481233	-2.3884203123	-0.5986822416
C39	2.5924764810	-1.2747779518	2.1741021003
C40	2.1459540712	-0.0260314721	1.3994296790
C41	0.6707260444#	-0.0054000452#	1.0951810226#
O42	0.4001196033	0.7392919198	0.0168461366
O43	-0.2204558187	-0.5417063581	1.7389425577
H44	-0.5303561684	-8.7202205479	5.2285235264
H45	-0.0250953887	-10.2351597511	-0.9829189331
H46	2.9539633773	-4.6473171717	-1.8133543272
H47	0.4896299319	-6.0480153402	7.8817411861
H48	0.9191361677	-7.6567899837	7.2941883493
H49	-0.7610566528	-7.1211571335	7.2577410955
H50	0.7925665457	-3.8269244453	7.2894440520
H51	1.3288016008	-2.8830853367	5.9107000699
H52	2.9958242850	-5.1767077481	7.0710441372
H53	3.1333794414	-3.5124038117	7.5843108763
H54	-1.3158578191	-10.7225839658	5.0044058690
H55	-1.7266783405	-11.9582698110	3.8256152973
H56	-2.8173362655	-10.5931865064	4.1070481985
H57	1.6653657575	-10.4532434842	-2.6411814634
H58	1.6932379664	-9.4932986176	-4.1151650850
H59	0.1667528203	-9.7756267370	-3.2874740058
H60	1.8067303981	-7.5861577816	-5.3079973218
H61	3.4077361811	-8.2225924109	-4.8905848053
H62	4.3151814682	-1.6281740707	-0.0614907822
H63	3.2608037938	-1.8914023268	-1.4525365737
H64	4.4545555290	-3.1156609104	-0.9977400326
H65	3.6840635902	-1.2336600467	2.2726468463
H66	2.2038273297	-1.2466423295	3.1941489469
H67	2.6872559051	0.0789497505	0.4556919967
H68	2.3663728663	0.8820264307	1.9777288029
C69	-3.2559239751#	-3.5334329856#	7.9400739519#
C70	-3.0744893311	-3.8924660346	6.4624273861
C71	-3.2914223406	-2.6980685519	5.5041771679
C72	-2.3616834032	-2.7273549299	4.2768112118
C73	-0.9418770866	-2.3191272010	4.7048491485
N74	0.0711141468	-2.4912197259	3.6541159083
H75	-2.0663285447	-4.3067534485	6.3205954700
H76	-3.7622176507	-4.7028749691	6.1881177343
H77	-4.3355780993	-2.6716933654	5.1730028083
H78	-3.1302356848	-1.7542970980	6.0448983229
H79	-2.3357527967	-3.7368550097	3.8446374884
H80	-2.7176645903	-2.0489165875	3.4927898381
H81	-0.9272212512	-1.2570291110	4.9690971179
H82	-0.6643833130	-2.8727851988	5.5952964578
C83	2.0373817063#	-12.3268851595#	0.0888090572#
C84	1.1796632147	-12.9129942950	1.1829434784

S85	-0.5414525007	-13.1399149366	0.6305379982
H86	1.4989408960	-13.9237006095	1.4577316442
H87	1.1552371242	-12.2963636030	2.0859709814
C88	6.8923719423	-6.8234938830	-2.5511133278
C89	6.8061860060	-6.9031267684	-1.0297591189
O90	6.5002398700	-5.9654623560	-0.2970195249
C91	5.5834903989	-7.2624598813	-3.2491657023
S92	4.4784863942	-5.8074456622	-3.3471983027
H93	5.8068940543	-7.6259842133	-4.2558740722
H94	5.0951130253	-8.0690351452	-2.6912144038
N95	7.0782809600	-8.1521666674	-0.5357121405
C96	7.1737736727#	-8.4196828939#	0.8846719061#
C97	5.8876016661	-8.9914759853	1.5281516675
C98	4.7284451715	-8.0472503223	1.6666538445
N99	4.8707487681	-6.7531107184	2.1493027542
C100	3.3768639765	-8.2205222056	1.5235177606
C101	3.6567665578	-6.1855954300	2.2566155616
N102	2.7180118015	-7.0550363715	1.8905771927
H103	7.4299958391	-8.8549621804	-1.1707138643
H104	7.4850656179	-7.4961540370	1.3823826608
H105	5.5559568280	-9.8646545214	0.9558686517
H106	6.1626874657	-9.3635868679	2.5260057221
H107	5.7382652573	-6.2300694869	2.1365692189
H108	2.8321096948	-9.0889334651	1.1920131914
H109	3.4992531481	-5.1733381157	2.5932222704
H110	5.6891195928	-3.6933155735	5.3381689679
H111	-0.5707947753	0.7738144600	-0.0910309282
H112	3.2651890366	-6.6997305990	-5.7760711344
H113	2.2423580524	-5.5732931283	-3.9040518405
H114	3.0858476435	-12.3135148607	0.4109002234
H115	1.7449051143	-11.3046248460	-0.1485724165
H116	1.9882008294	-12.9289876021	-0.8243016289
H117	7.1519027620	-5.7975201580	-2.8270176644
H118	7.7079035616	-7.4647787729	-2.9068912885
H119	-4.2561200874	-3.1283607563	8.1317214642
H120	-2.5298247915	-2.7733934847	8.2533179187
H121	-3.1264244623	-4.4093856497	8.5852118812
H122	-3.1260682173	-13.0358926823	0.9280876374
H123	-3.3093136810	-11.3571927340	0.3504670171
H124	-3.3094439744	-11.6819946512	2.0683572123
H125	7.9751545415	-9.1480223311	1.0425749420
H126	0.0596486341	-1.7287487656	2.9632129818
H127	-0.9638484344	-6.5364861346	-0.6979884716
N128	-1.6024760810	-6.2353149297	0.0431967008
H129	-2.4868289160	-5.7724834895	-0.1575819066
O130	-1.0703320641	-5.9584835923	1.1831394871

The structure in **Figure S4**

Energies: E= -3896.317549, solv = -0.149953, disp = -170.70 Z_0 = 668.65

Fe1	0.7658632549	-6.5306434494	1.5131944871
C2	0.7590013622	-3.6047237827	3.2453847946
C3	-0.2081900550	-8.1640398061	4.3276209282
C4	0.2785471692	-9.3723267178	-0.3431036339
C5	2.1559048496	-5.0057194290	-1.1460083659
N6	0.5574587598	-6.0129693889	3.4484984283
C7	0.7720523102	-4.8226736499	4.0295415573
C8	0.8530362979	-4.9164758527	5.4647077450
C9	0.4841594823	-6.2104233320	5.7573580665
C10	0.2961899764	-6.8913698260	4.4940793409
C11	0.2743430849#	-6.7888710181#	7.1082149257#
C12	1.4170914344	-3.8927006916	6.4218162804
C13	2.8930388151	-4.1483759802	6.7732575961
C14	3.8297630218#	-3.8698639823#	5.6169420360#
O15	5.0970800093	-4.1876053970	5.9341067939
O16	3.5165409979	-3.4046068933	4.5354263899
N17	0.1535569379	-8.4492440240	1.9043887561
C18	-0.3815480206	-8.8573845386	3.1126545353
C19	-1.0549589890	-10.0991277535	2.9676467118
C20	-0.8241366642	-10.5271453517	1.6496635163
C21	-0.0605046959	-9.4472100977	1.0159713386
C22	-1.7618710645#	-10.8824638913#	4.0177929561#
C23	-1.3864206809	-11.7301612589	1.0976014912
C24	-2.8547534254#	-11.9954430124#	1.1219388761#
N25	1.1307059382	-7.0751653571	-0.3669763382
C26	0.8907871833	-8.2926776456	-0.9508797521
C27	1.4089285951	-8.3303484410	-2.3120876020
C28	1.9904669207	-7.1044232389	-2.5368702434
C29	1.7599725968	-6.3332489582	-1.3140168488
C30	1.2334229058#	-9.5722280290#	-3.1310109934#
C31	2.7273715058	-6.4879516829	-3.7064202222
C32	2.8164840743#	-7.3065219800#	-4.9860737278#
N33	1.4014110754	-4.6528294598	1.1537824918
C34	2.0461965040	-4.2487911796	0.0039910058
C35	2.6052566370	-2.9318701517	0.1685233381
C36	2.2219291032	-2.5055975369	1.4264874118
C37	1.4444312501	-3.5825218959	1.9773137146
C38	3.4798127672	-2.2287109986	-0.8259357475
C39	2.6358631723	-1.2316964154	2.1176903922
C40	2.1458617805	0.0336680356	1.3955324752
C41	0.6707260553#	-0.0054000680#	1.0951810334#
O42	0.3623141744	0.6875866108	-0.0032255894
O43	-0.1927530624	-0.5534849433	1.7686538631
H44	-0.5323749106	-8.6753900499	5.2280851351
H45	0.0581009569	-10.2335958018	-0.9608874330
H46	2.6195180221	-4.5120868598	-1.9853465838
H47	0.2577463681	-6.0126409416	7.8773811623

H48	1.0587225198	-7.5062217753	7.3848593809
H49	-0.6813969887	-7.3235719317	7.1695027049
H50	0.8376498283	-3.8871151640	7.3519587032
H51	1.3559385180	-2.8859515763	6.0060850600
H52	3.0639887119	-5.1729252380	7.1217480776
H53	3.1993176444	-3.4967915093	7.6019457102
H54	-1.4203770199	-10.6212440990	5.0235177705
H55	-1.6055323666	-11.9590815302	3.8993800022
H56	-2.8434659986	-10.6973666009	3.9994022080
H57	1.7319390492	-10.4314941651	-2.6662869454
H58	1.6360738553	-9.4712426182	-4.1376433732
H59	0.1705441271	-9.8217441591	-3.2335159132
H60	1.8164241089	-7.5689879513	-5.3485435767
H61	3.3837810407	-8.2321997692	-4.8498825482
H62	4.1333365370	-1.5038847799	-0.3314666294
H63	2.8893848413	-1.6818680702	-1.5728011971
H64	4.1238040912	-2.9315628080	-1.3633359485
H65	3.7304813836	-1.1898768140	2.1702088378
H66	2.2975978119	-1.2338621179	3.1558494325
H67	2.6813864806	0.2008637286	0.4583369782
H68	2.3280280502	0.9222396962	2.0154086423
C69	-3.2559239755#	-3.5334329756#	7.9400739341#
C70	-3.1073522662	-3.7431611620	6.4286338385
C71	-3.1740755460	-2.4319707102	5.6111395372
C72	-2.3123084243	-2.4559132191	4.3319417890
C73	-0.8338553291	-2.3320671651	4.7389325952
N74	0.1034191633	-2.5253270494	3.6236626738
H75	-2.1550362381	-4.2592036218	6.2386299966
H76	-3.8847840094	-4.4286087354	6.0682385135
H77	-4.2138722139	-2.2178534995	5.3409571281
H78	-2.8510388812	-1.5872708068	6.2369303417
H79	-2.4643873844	-3.3944689829	3.7834832561
H80	-2.5843844166	-1.6380761188	3.6556220564
H81	-0.6387196186	-1.3320712685	5.1409145988
H82	-0.6261520207	-3.0541795784	5.5182223608
C83	2.0373816851#	-12.3268851664#	0.0888090640#
C84	1.1268006937	-12.9980061643	1.0831839483
S85	-0.5828095280	-13.0847488881	0.4644701864
H86	1.3939516568	-14.0485069726	1.2398992831
H87	1.1047972738	-12.4958528902	2.0544153371
C88	6.8704713480	-6.8254126498	-2.5659638137
C89	6.8506619512	-6.8896736242	-1.0416863303
O90	6.6721290088	-5.9054123607	-0.3193090058
C91	5.5341031761	-7.2542628245	-3.2185192491
S92	4.4121108725	-5.8060308073	-3.2364940955
H93	5.7214129049	-7.5838924735	-4.2439011513
H94	5.0761993596	-8.0826783162	-2.6668684721
N95	7.0312066139	-8.1442406906	-0.5358833395
C96	7.1737736386#	-8.4196828766#	0.8846718958#
C97	5.9155486818	-8.9962827823	1.5633927828

C98	4.7602072962	-8.0433634216	1.6496687347
N99	4.9504571107	-6.6754077356	1.7621954766
C100	3.4035433086	-8.2254550810	1.7398966998
C101	3.7440902539	-6.0913624126	1.8882432351
N102	2.7729659585	-6.9984672156	1.8822041225
H103	7.2572256704	-8.8894150471	-1.1805554742
H104	7.4988940126	-7.4975193164	1.3730295442
H105	5.5986722682	-9.9056074448	1.0399582636
H106	6.2097143563	-9.3107267639	2.5752765661
H107	5.7721106764	-6.1898764691	1.3861949872
H108	2.8384976112	-9.1436751046	1.7237610594
H109	3.6234767461	-5.0260122662	1.9759594500
H110	5.6630731243	-3.9387386971	5.1784971598
H111	-0.6098397202	0.6746636054	-0.1101982332
H112	3.3109045651	-6.7201601816	-5.7647643729
H113	2.2120036604	-5.5519672726	-3.9572594139
H114	3.0798387742	-12.4313660487	0.4135531505
H115	1.8219749107	-11.2625959343	-0.0031274287
H116	1.9607396287	-12.7878594754	-0.9014021976
H117	7.1321850895	-5.8046701795	-2.8576204428
H118	7.6641786627	-7.4803576410	-2.9456947995
H119	-4.2018800340	-3.0356916437	8.1817337061
H120	-2.4464055476	-2.9064381262	8.3335782092
H121	-3.2384626176	-4.4865163875	8.4798177842
H122	-3.1340871922	-12.8210904065	0.4604003945
H123	-3.4244389213	-11.0988885340	0.8584397211
H124	-3.1605418295	-12.2885942369	2.1362530963
H125	7.9881348259	-9.1404142900	1.0107485247
H126	0.0807928318	-1.7507536183	2.9352500416
N127	-1.7474884870	-6.8051808875	0.4874183145
O128	-0.9295683140	-5.9625231070	1.2094949890
N129	-2.8980847421	-6.3378736348	0.4052829706
O130	-3.4182496921	-5.3060283536	0.8280304375

The structure of NH_2OH bound to the oxo on cyt-P460
Energies: E= -3843.442225, solv = -0.154020, disp = -172.13 Z_0 = 688.14

Fe1	0.7093185406	-6.5087168190	1.5069295459
C2	0.7413747093	-3.6043715411	3.2496222007
C3	-0.2182845584	-8.1661750150	4.3340785002
C4	0.2623837889	-9.3543645342	-0.3430551596
C5	2.2039042725	-5.0158068378	-1.1257387699
N6	0.5298828128	-6.0119743854	3.4495794495
C7	0.7590755339	-4.8242913164	4.0299101022
C8	0.8549373956	-4.9228616382	5.4638949401
C9	0.4796152115	-6.2135250050	5.7567775868
C10	0.2791070561	-6.8916354202	4.4956328294
C11	0.2743431327#	-6.7888710444#	7.1082149075#
C12	1.4215476264	-3.9040475681	6.4235498718

C13	2.8970008183	-4.1600493387	6.7726762906
C14	3.8297630053#	-3.8698639759#	5.6169420443#
O15	5.1015293560	-4.1672014931	5.9354983598
O16	3.5102764817	-3.4130508256	4.5335973305
N17	0.1476783535	-8.4446335517	1.9138281180
C18	-0.3884045370	-8.8557063570	3.1185957519
C19	-1.0605382214	-10.0963523657	2.9685549693
C20	-0.8344756575	-10.5160832914	1.6484849483
C21	-0.0735139550	-9.4336553646	1.0173567388
C22	-1.7618710626#	-10.8824638979#	4.0177929531#
C23	-1.3867704986	-11.7302892394	1.1065296382
C24	-2.8547534063#	-11.9954430220#	1.1219388665#
N25	1.1655755606	-7.0768821036	-0.3577515119
C26	0.8999967225	-8.2865942144	-0.9454722476
C27	1.4199496553	-8.3297804815	-2.3087589769
C28	2.0171882353	-7.1103018165	-2.5292823849
C29	1.8009300182	-6.3406061905	-1.3019812527
C30	1.2334228973#	-9.5722280379#	-3.1310109917#
C31	2.7427085527	-6.4905257664	-3.7045317359
C32	2.8164840700#	-7.3065219640#	-4.9860736611#
N33	1.4106935868	-4.6629803862	1.1635058925
C34	2.0731656497	-4.2619052554	0.0223517790
C35	2.6228794648	-2.9421106906	0.1930458934
C36	2.2129846922	-2.5118674423	1.4403646072
C37	1.4246876632	-3.5866065442	1.9804479636
C38	3.5142886374	-2.2404515699	-0.7869149965
C39	2.6209116236	-1.2400606208	2.1396830891
C40	2.1396232693	0.0321664168	1.4223305554
C41	0.6707260592#	-0.0054000801#	1.0951810558#
O42	0.3883593573	0.6588967356	-0.0255762267
O43	-0.2068125207	-0.5342518587	1.7693485241
H44	-0.5371656012	-8.6796032782	5.2350428743
H45	0.0285129025	-10.2100641867	-0.9640655604
H46	2.6803635296	-4.5210704602	-1.9566807235
H47	0.2479905721	-6.0098433528	7.8743160103
H48	1.0662679995	-7.4961148684	7.3897382073
H49	-0.6753406032	-7.3339318011	7.1710894357
H50	0.8421373763	-3.9020472971	7.3536880995
H51	1.3592234655	-2.8963557373	6.0129535921
H52	3.0709268308	-5.1875315147	7.1111248637
H53	3.2031432475	-3.5157114241	7.6069697480
H54	-1.3982945154	-10.6408958734	5.0208596760
H55	-1.6253434113	-11.9598144184	3.8838423263
H56	-2.8404279045	-10.6792179808	4.0224193725
H57	1.7163841901	-10.4369086785	-2.6602618581
H58	1.6474240572	-9.4760240906	-4.1333768364
H59	0.1681777974	-9.8070673682	-3.2431147442
H60	1.8124168783	-7.5692377063	-5.3372158594
H61	3.3863860339	-8.2319642544	-4.8582641864
H62	4.1506094136	-1.5060745060	-0.2840101169

H63	2.9344783940	-1.7052211727	-1.5502600510
H64	4.1756284771	-2.9420459249	-1.3045944009
H65	3.7150555821	-1.1991785014	2.1998560257
H66	2.2766253103	-1.2473301066	3.1756236002
H67	2.6897529094	0.2086706440	0.4954880431
H68	2.3108158554	0.9136844513	2.0551889361
C69	-3.2559239665#	-3.5334329450#	7.9400738747#
C70	-3.2077440216	-3.5176383868	6.4066526594
C71	-3.0390571759	-2.1006751472	5.8085656332
C72	-2.2400071609	-2.0641077250	4.4884113505
C73	-0.7636734667	-2.3278557291	4.8198279472
N74	0.0978507286	-2.5238814109	3.6451392526
H75	-2.3875833345	-4.1714020053	6.0763804861
H76	-4.1196881819	-3.9719637832	6.0000230587
H77	-4.0248368836	-1.6521012089	5.6437650978
H78	-2.5375177739	-1.4494186403	6.5396858275
H79	-2.6006710262	-2.8169201417	3.7779980101
H80	-2.3454821453	-1.0877051726	4.0011466516
H81	-0.3546546428	-1.4813059833	5.3842908869
H82	-0.7012797017	-3.2111787845	5.4444651061
C83	2.0373816651#	-12.3268851729#	0.0888090684#
C84	1.1422177847	-12.9678103489	1.1174499573
S85	-0.5703717282	-13.0928710349	0.5145455482
H86	1.4205139476	-14.0090897902	1.3116065372
H87	1.1228960020	-12.4282674452	2.0684893931
C88	6.8803581556	-6.8241547195	-2.5641695152
C89	6.8499793429	-6.8881676068	-1.0398504904
O90	6.6741861516	-5.9036306674	-0.3178117549
C91	5.5497947862	-7.2562646793	-3.2256763969
S92	4.4308375558	-5.8063971977	-3.2585240649
H93	5.7438692744	-7.5912789599	-4.2481022985
H94	5.0860801305	-8.0807721112	-2.6730888858
N95	7.0191948231	-8.1448856070	-0.5346701430
C96	7.1737736061#	-8.4196828597#	0.8846718823#
C97	5.9290853926	-9.0052428920	1.5750526925
C98	4.7792988694	-8.0505533037	1.6823285100
N99	4.9753900890	-6.6837916771	1.7959132483
C100	3.4234667196	-8.2282338314	1.7904217117
C101	3.7683372820	-6.0995816140	1.9372691452
N102	2.7965603940	-7.0024744194	1.9426290101
H103	7.2480368356	-8.8891120579	-1.1794556896
H104	7.4965973408	-7.4953867912	1.3702278111
H105	5.6056911552	-9.9106480406	1.0486160999
H106	6.2384307379	-9.3276632707	2.5799294269
H107	5.7924600662	-6.1980285631	1.4129295148
H108	2.8569363533	-9.1462761518	1.7781361178
H109	3.6499729591	-5.0340201671	2.0318367006
H110	5.6635948918	-3.9178892899	5.1770914646
H111	-0.5794313628	0.6420949396	-0.1631657678
H112	3.3012979357	-6.7186584914	-5.7696397080

H113	2.2219718707	-5.5550619018	-3.9466027340
H114	3.0833074777	-12.4061788115	0.4089326152
H115	1.8087286667	-11.2692951005	-0.0431955019
H116	1.9583297166	-12.8281055795	-0.8813812812
H117	7.1417296931	-5.8028324736	-2.8539070422
H118	7.6788851598	-7.4769864277	-2.9375304690
H119	-4.0723527997	-2.9084907592	8.3192452456
H120	-2.3224772454	-3.1478432930	8.3687706812
H121	-3.4116101269	-4.5473811861	8.3243133287
H122	-3.1252529709	-12.8705793857	0.5240743313
H123	-3.4159255113	-11.1205279763	0.7773797564
H124	-3.1818437942	-12.1988723627	2.1510622437
H125	7.9962348439	-9.1329203731	1.0032770994
H126	0.0634791168	-1.7446491460	2.9652866443
O127	-0.8221570236	-6.1103724633	1.1311634539
H128	-1.2586987831	-4.3964540298	0.4499872607
O129	-1.3780803050	-3.4965633113	0.0882765129
N130	-2.3095018317	-2.8991617224	1.0176855701
H131	-1.9482137977	-1.9490861850	1.1176543782
H132	-3.1769875319	-2.8055726846	0.4823507495

The structure of NH_2O bound to OH on cyt-P460
Energies: E= -3843.459689, solv = -0.153559, disp = -172.86 Z_0 = 686.38

Fe1	0.7445550275	-6.5281163250	1.5069602354
C2	0.7509004218	-3.6114984966	3.2482624194
C3	-0.2128811947	-8.1707829199	4.3256202124
C4	0.2900416929	-9.3618901104	-0.3409218938
C5	2.1576638439	-4.9939700222	-1.1505513909
N6	0.5561798445	-6.0196643247	3.4476911569
C7	0.7775782906	-4.8292672112	4.0290989832
C8	0.8591537620	-4.9270453082	5.4652344369
C9	0.4831653172	-6.2185036824	5.7550206844
C10	0.2927543108	-6.8971303958	4.4909914703
C11	0.2743431373#	-6.7888710499#	7.1082149103#
C12	1.4275006169	-3.9141707611	6.4309372079
C13	2.9032708618	-4.1776184460	6.7732856465
C14	3.8297630050#	-3.8698639746#	5.6169420445#
O15	5.0904181580	-4.2507503520	5.8902653095
O16	3.5153260209	-3.3309120338	4.5711213041
N17	0.1505011665	-8.4531497127	1.9071951340
C18	-0.3854742166	-8.8652967525	3.1113271863
C19	-1.0594038067	-10.1048960735	2.9632111327
C20	-0.8249705229	-10.5322569349	1.6429078683
C21	-0.0543378245	-9.4435849971	1.0184230961
C22	-1.7618710631#	-10.8824639006#	4.0177929543#

C23	-1.3922189683	-11.7140050418	1.0678891973
C24	-2.8547534036#	-11.9954430242#	1.1219388645#
N25	1.1703227643	-7.0743247214	-0.3618415540
C26	0.9157468246	-8.2886810768	-0.9450890842
C27	1.4244589493	-8.3288741187	-2.3119730353
C28	2.0078100230	-7.1038408805	-2.5379560464
C29	1.7851830005	-6.3310367284	-1.3131661462
C30	1.2334228965#	-9.5722280392#	-3.1310109935#
C31	2.7382241188	-6.4862899058	-3.7087948454
C32	2.8164840702#	-7.3065219628#	-4.9860736556#
N33	1.3639480110	-4.6437766113	1.1352242978
C34	2.0176999175	-4.2315260842	-0.0064302775
C35	2.5788303307	-2.9139841791	0.1708370967
C36	2.1956143108	-2.5017307229	1.4317661592
C37	1.4114057356	-3.5837697935	1.9686720325
C38	3.4611923530	-2.2039079105	-0.8115640834
C39	2.6173899989	-1.2462502205	2.1515269659
C40	2.1281918794	0.0419754347	1.4678083283
C41	0.6707260593#	-0.0054000779#	1.0951810586#
O42	0.4308452223	0.6431905398	-0.0468189068
O43	-0.2353072070	-0.5203052731	1.7440364259
H44	-0.5370348059	-8.6801843172	5.2272108999
H45	0.0693882809	-10.2213616698	-0.9611122756
H46	2.6271040336	-4.5001761356	-1.9870922960
H47	0.2340720433	-6.0065539614	7.8703747322
H48	1.0719618457	-7.4860168688	7.3990635862
H49	-0.6689397332	-7.3454511131	7.1675401199
H50	0.8500915911	-3.9164491382	7.3623630714
H51	1.3702308863	-2.9037887883	6.0263813579
H52	3.0772114871	-5.2087603688	7.0987602306
H53	3.2134530161	-3.5416112214	7.6129970183
H54	-1.4181915976	-10.6166469342	5.0215690779
H55	-1.6066251294	-11.9599916639	3.9036996814
H56	-2.8444136266	-10.7005596515	4.0003430162
H57	1.7253372804	-10.4350342582	-2.6661241546
H58	1.6333298903	-9.4760158575	-4.1392700830
H59	0.1677068704	-9.8112716241	-3.2282067886
H60	1.8130458220	-7.5647665072	-5.3423215788
H61	3.3790382058	-8.2351241368	-4.8503433634
H62	4.1024879049	-1.4736224038	-0.3089678808
H63	2.8760680911	-1.6619667594	-1.5661473383
H64	4.1178469538	-2.9011917039	-1.3410479870
H65	3.7121026318	-1.2047503951	2.1983738508
H66	2.2887051667	-1.2766481810	3.1917209943
H67	2.6976790081	0.2591405942	0.5616983094
H68	2.2655280663	0.9048353506	2.1339803846
C69	-3.2559239655#	-3.5334329393#	7.9400738670#
C70	-3.2058655633	-3.5159162326	6.4081599346
C71	-3.0263204937	-2.0993179343	5.8138438265
C72	-2.2228238626	-2.0768359457	4.4970504525

C73	-0.7444091980	-2.3274688543	4.8275817596
N74	0.1114802720	-2.5250802496	3.6512836192
H75	-2.3892140063	-4.1735606243	6.0775360365
H76	-4.1208155468	-3.9628183425	5.9995462580
H77	-4.0078534504	-1.6415715364	5.6484622483
H78	-2.5183688591	-1.4525420937	6.5439583308
H79	-2.5837777720	-2.8544419456	3.8125793484
H80	-2.3372953048	-1.1128308399	3.9870109764
H81	-0.3441960134	-1.4757672503	5.3901878242
H82	-0.6773858463	-3.2071572708	5.4567465901
C83	2.0373816621#	-12.3268851744#	0.0888090687#
C84	1.0863393175	-13.0429062175	1.0076716874
S85	-0.6088274900	-13.0432685058	0.3445408398
H86	1.3242122138	-14.1083566446	1.0960643689
H87	1.0510129181	-12.6111413386	2.0116557240
C88	6.8776125952	-6.8562195973	-2.5802813094
C89	6.8593926961	-6.9057656597	-1.0555253538
O90	6.7063501003	-5.9097912140	-0.3432815505
C91	5.5351285238	-7.2710262205	-3.2279657437
S92	4.4274094491	-5.8119466563	-3.2458035944
H93	5.7159789693	-7.6048600121	-4.2531783762
H94	5.0691749171	-8.0929785521	-2.6734088814
N95	7.0118561376	-8.1586737712	-0.5368348449
C96	7.1737736021#	-8.4196828568#	0.8846718811#
C97	5.9285434688	-8.9889576322	1.5897833542
C98	4.7754641765	-8.0344695095	1.6733641405
N99	4.9661556035	-6.6633544721	1.7298312177
C100	3.4206867474	-8.2132278542	1.7973682464
C101	3.7580571827	-6.0785011164	1.8532820611
N102	2.7896750332	-6.9843853162	1.9018253408
H103	7.2163088438	-8.9155910385	-1.1750413618
H104	7.5035986618	-7.4908543741	1.3569013804
H105	5.6063310528	-9.9077936472	1.0862094959
H106	6.2393842699	-9.2868271558	2.6017515594
H107	5.7836333293	-6.1879586118	1.3319070080
H108	2.8572797034	-9.1327718345	1.8247337268
H109	3.6353178733	-5.0106175562	1.8991487864
H110	5.6501341274	-3.9794477776	5.1376425760
H111	-0.5315401972	0.6423721494	-0.2145577861
H112	3.3103010196	-6.7257487729	-5.7694241312
H113	2.2249331548	-5.5482975993	-3.9563390501
H114	3.0686084876	-12.4742427203	0.4327732877
H115	1.8453765097	-11.2540729135	0.0634996874
H116	1.9792038621	-12.7173676446	-0.9325571727
H117	7.1531981382	-5.8417402291	-2.8806937213
H118	7.6617534555	-7.5258370439	-2.9545247486
H119	-4.0676002369	-2.9026517227	8.3199082521
H120	-2.3195840582	-3.1564678467	8.3698211376
H121	-3.4197278698	-4.5466433808	8.3224837887
H122	-3.1538474088	-12.7231276207	0.3604331424

H123	-3.4463834574	-11.0823222581	1.0111453367
H124	-3.1123204082	-12.4456045800	2.0914561829
H125	7.9924546167	-9.1366009605	1.0070661881
H126	0.0681496431	-1.7544795216	2.9676045401
O127	-0.9582774476	-6.1922405979	1.1339827973
H128	-1.0529067225	-5.2534430358	0.8706806491
O129	-1.2752611736	-3.2778280536	0.6080046785
N130	-2.3044583053	-2.5581474439	0.8712986782
H131	-2.1406275498	-1.5724327769	1.0819398141
H132	-3.2166651601	-2.8576808214	0.5246768401

The structure of NHO bound to H_2O on cyt-P460
Energies: E= -3843.468611, solv = -0.147842, disp = -175.47 Z_0 =686.45

Fe1	0.8656538601	-6.6021368938	1.5277570220
C2	0.6963536137	-3.6349795817	3.2529336570
C3	-0.1918374071	-8.2356311244	4.3206719869
C4	0.3413218014	-9.4066395407	-0.3602710271
C5	2.2394447095	-5.0434642396	-1.1191250085
N6	0.5158568052	-6.0676859906	3.4222445096
C7	0.7244852783	-4.8606772997	4.0042505269
C8	0.8371694538	-4.9626303416	5.4305853376
C9	0.4746066713	-6.2567106734	5.7344179898
C10	0.2818514599	-6.9481670346	4.4774209550
C11	0.2743431481#	-6.7888710488#	7.1082148906#
C12	1.4061098526	-3.9403695928	6.3862746404
C13	2.8782690399	-4.2049162732	6.7468445141
C14	3.8297630051#	-3.8698639736#	5.6169420432#
O15	5.0937757476	-4.2029673605	5.9383886208
O16	3.5331488142	-3.3523218741	4.5562366639
N17	0.2140313638	-8.4967619381	1.8916886736
C18	-0.3393497953	-8.9249744767	3.0999687669
C19	-1.0307444449	-10.1425901507	2.9416728567
C20	-0.8131459255	-10.5595610494	1.5907606861
C21	0.0005472044	-9.4830057858	0.9814682000
C22	-1.7618710929#	-10.8824638929#	4.0177929776#
C23	-1.4333589151	-11.6172412002	0.9057963202
C24	-2.8547532628#	-11.9954430329#	1.1219388285#
N25	1.1792619571	-7.1027053832	-0.3552847529
C26	0.9519495528	-8.3095556660	-0.9475317051
C27	1.4626355452	-8.3359345783	-2.3085455892
C28	2.0652638560	-7.1149181392	-2.5211696180
C29	1.8508030392	-6.3436563063	-1.2995323134
C30	1.2334228837#	-9.5722280333#	-3.1310109806#
C31	2.7720870737	-6.4902703971	-3.7058518464
C32	2.8164840657#	-7.3065219590#	-4.9860736233#

N33	1.3655811401	-4.6984960598	1.1492898483
C34	2.0630926872	-4.2876290111	0.0438687546
C35	2.6091432252	-2.9815367534	0.2352194880
C36	2.1612814581	-2.5493661847	1.4749444844
C37	1.3680136426	-3.6149250818	2.0004046404
C38	3.5375547997	-2.2770748981	-0.7099633194
C39	2.5785123386	-1.2865136178	2.1844435686
C40	2.1255358254	-0.0016820474	1.4752085437
C41	0.6707260666#	-0.0054000693#	1.0951810599#
O42	0.4300454903	0.8096698936	0.0648973688
O43	-0.2333093420	-0.6288841828	1.6445406444
H44	-0.5129679293	-8.7501963119	5.2194187905
H45	0.1480450856	-10.2648160349	-0.9826993695
H46	2.7557204125	-4.5472022978	-1.9246546147
H47	0.1593015797	-5.9794102755	7.8332158266
H48	1.1130677978	-7.4135525441	7.4448490524
H49	-0.6260128909	-7.4116013066	7.1699369480
H50	0.8215596425	-3.9243117337	7.3131514412
H51	1.3583372099	-2.9375947034	5.9640847561
H52	3.0533515656	-5.2409485314	7.0567598781
H53	3.1692257195	-3.5850335068	7.6049638825
H54	-1.3116243879	-10.7180377879	5.0007140271
H55	-1.7639394038	-11.9600771164	3.8333443225
H56	-2.8086166317	-10.5587287923	4.0849720447
H57	1.7117373916	-10.4458388050	-2.6729222752
H58	1.6239820929	-9.4830655782	-4.1439003833
H59	0.1621482669	-9.7898149844	-3.2122428331
H60	1.8055602121	-7.5723763744	-5.3152801078
H61	3.3894698847	-8.2316019644	-4.8687227777
H62	4.1590509986	-1.5485987549	-0.1802037616
H63	2.9939149352	-1.7337692034	-1.4942714333
H64	4.2149780316	-2.9789124639	-1.2060905746
H65	3.6729032616	-1.2617798889	2.2482666095
H66	2.2316451591	-1.2974118008	3.2187257448
H67	2.7028706222	0.1864383687	0.5662077576
H68	2.2896448228	0.8731554903	2.1196720337
C69	-3.2559239666#	-3.5334329332#	7.9400738615#
C70	-3.2969225871	-3.4778224905	6.4076197056
C71	-3.0681960567	-2.0581788027	5.8316341471
C72	-2.2395872459	-2.0377036435	4.5303315780
C73	-0.7785347666	-2.3600805567	4.8726760285
N74	0.0938695820	-2.5289128841	3.7041068361
H75	-2.5434838831	-4.1740177044	6.0115341026
H76	-4.2601136058	-3.8608611619	6.0488120275
H77	-4.0331367369	-1.5713298096	5.6534940416
H78	-2.5563904495	-1.4338925707	6.5783260878
H79	-2.6240453365	-2.7776966667	3.8167713677
H80	-2.3059027420	-1.0550680640	4.0468656387
H81	-0.3655066498	-1.5590886075	5.4977571765
H82	-0.7561860274	-3.2751408842	5.4550736496

C83	2.0373815714#	-12.3268852085#	0.0888090632#
C84	0.8413668415	-13.2195885358	0.3141998712
S85	-0.7681717634	-12.6239432557	-0.3616243857
H86	0.9557283401	-14.1781532385	-0.2033877958
H87	0.6780925403	-13.4360698704	1.3731325085
C88	6.9024169432	-6.8102604663	-2.5567180000
C89	6.8562375436	-6.8843394952	-1.0338942138
O90	6.6636895859	-5.9075145558	-0.3039858605
C91	5.5823831096	-7.2517200933	-3.2360283913
S92	4.4685500621	-5.7993774137	-3.3126752459
H93	5.7954928023	-7.6081706636	-4.2473315917
H94	5.1061312100	-8.0647845557	-2.6771717665
N95	7.0334461060	-8.1420497972	-0.5344704316
C96	7.1737735819#	-8.4196828485#	0.8846718793#
C97	5.9090448875	-8.9797817444	1.5685061420
C98	4.7547980111	-8.0237967198	1.6539493055
N99	4.9393165013	-6.6529086635	1.7710958381
C100	3.4009643322	-8.2151686980	1.7375616085
C101	3.7371399600	-6.0675824220	1.8952034782
N102	2.7712733339	-6.9859384860	1.8820675470
H103	7.2689533961	-8.8826580944	-1.1810380065
H104	7.5114746609	-7.5018994592	1.3735224212
H105	5.5829975867	-9.8896613870	1.0523509907
H106	6.2008636505	-9.2894150831	2.5823802867
H107	5.7648018214	-6.1674209026	1.3993738371
H108	2.8345197219	-9.1312015035	1.7103705139
H109	3.6067498625	-5.0032033116	1.9805975538
H110	5.6708108040	-3.9116495727	5.2067395772
H111	-0.5329928265	0.8377413469	-0.0972357742
H112	3.2839226212	-6.7209736117	-5.7817726643
H113	2.2451764080	-5.5545264987	-3.9329170745
H114	2.9383402023	-12.8382022182	0.4496396620
H115	1.9461801992	-11.3812932264	0.6261952928
H116	2.1832670429	-12.1097315468	-0.9738026381
H117	7.1573402797	-5.7847998782	-2.8373817910
H118	7.7124196069	-7.4527660072	-2.9231364028
H119	-4.0069822769	-2.8695314451	8.3826545472
H120	-2.2760325593	-3.2186829883	8.3205599226
H121	-3.4530849742	-4.5459833805	8.3084623875
H122	-3.3430324692	-12.1351933966	0.1475653906
H123	-3.4134425842	-11.2430353598	1.6838016249
H124	-2.9412412727	-12.9652796355	1.6305985902
H125	7.9767524427	-9.1527268843	1.0108859949
H126	0.0622467817	-1.7532252428	3.0336267225
O127	-1.0792259105	-6.2189432598	1.0599309491
H128	-1.1933196574	-5.2937322963	0.7266592813
O129	-1.3789008869	-3.5923467586	0.2044559729
N130	-2.0538762552	-2.9725951566	1.0034051201
H131	-1.9175076567	-1.9445085623	0.8140923074
H132	-1.3005572535	-6.8013301590	0.3129050823

The structure in **Figure S5**

Energies: E= -4011.400767, solv = -0.074412, disp = -181.41 Z_0 = 684.20

Fe1	10.0463410000	48.8893680000	25.5677570000
N2	8.1017080000	48.6209570000	25.8439820000
N3	9.9657860000	50.3994540000	26.8083620000
N4	12.0464910000	49.2344320000	25.2742260000
N5	10.1060360000	47.3080390000	24.3128730000
C6	7.4311580000	47.4484540000	25.6520830000
O7	2.4580000457#	47.4399990107#	26.0699998932#
C8	8.8945860000	51.1586420000	27.0750840000
C9	12.8702750000	50.0674360000	25.9243770000
C10	11.1254340000	46.9344110000	23.4754200000
O11	9.1368390000	40.8446150000	22.3519280000
C12	6.0717690000	47.5548370000	26.1838370000
O13	3.2791130000	48.1266420000	24.1154560000
C14	9.2522460000	52.3729620000	27.8677760000
C15	14.1899060000	50.1668770000	25.2837260000
C16	10.7693260000	45.7041520000	22.7542090000
O17	7.2990000322#	41.4389989948#	23.5170000184#
C18	5.8698020000	48.8649490000	26.5452520000
C19	10.5675850000	52.2313650000	28.1690970000
C20	14.1193080000	49.2996540000	24.2045300000
C21	9.5281360000	45.3438000000	23.2412810000
C22	7.1975380000	49.5080120000	26.3764310000
C23	11.0368770000	50.8472740000	27.6981510000
C24	12.7839920000	48.6896190000	24.2162170000
C25	9.1320890000	46.3561490000	24.1993520000
C26	5.1748070000	46.3522520000	26.3060470000
C27	11.4429660000	53.0225800000	29.0757710000
C28	15.1876410000	48.9555590000	23.2044150000
C29	8.7464790000	44.1137530000	22.8744600000
C30	4.2329830000	46.1281110000	25.0957050000
C31	10.6800002026#	53.7659989469#	30.1639996707#
C32	16.5419997721#	48.6100011805#	23.8370000613#
C33	9.1953650000	42.8667150000	23.6843520000
C34	3.2779630000	47.3002590000	24.9917170000
C35	8.5831030000	41.6041960000	23.1133620000
C36	7.9152260000	46.3862780000	24.8937220000
C37	7.5575610000	50.7814110000	26.8004100000
C38	12.5432520000	50.7028510000	27.2474020000
C39	12.3510680000	47.6433330000	23.4107700000
C40	4.5840006224#	49.5429986522#	27.0259989254#
C41	8.2430004571#	53.4420002595#	28.2979997012#
C42	15.4039996068#	51.0260007424#	25.7099992017#
C43	11.5399995419#	44.9049995398#	21.7090011437#
C44	12.9969999464#	54.0449979387#	25.3990003895#

C45	12.0346850000	54.5028590000	26.5043530000
S46	12.7001880000	54.1293090000	28.1932400000
N47	13.2501910000	52.4936490000	22.4362180000
C48	13.3028980000	51.8699820000	21.1104570000
C49	11.8756230000	51.8479790000	20.5368290000
C50	13.8952890000	50.4431470000	21.0805940000
S51	15.5175070000	50.3008630000	21.9450720000
N52	11.3286870000	53.2010060000	20.5164480000
C53	9.8846080000	53.3311540000	20.5667010000
C54	9.2189500000	53.2332720000	21.9756100000
C55	9.2636180000	51.9015050000	22.6573060000
N56	8.5945130000	50.7879920000	22.1700720000
C57	9.8769800000	51.4558070000	23.8032680000
C58	8.8171810000	49.7422520000	22.9966950000
N59	9.5940640000	50.1151890000	24.0035360000
C60	16.9891350068#	48.9429349524#	31.0169701364#
C61	16.1410480000	48.1334430000	30.0155290000
C62	14.7403720000	48.6710260000	29.7713780000
C63	13.6976560000	48.4069920000	30.6750440000
C64	14.4498660000	49.4269550000	28.6234050000
C65	12.3917450000	48.8526910000	30.4575860000
C66	13.1523810000	49.8795070000	28.3899070000
C67	12.1423060000	49.5705510000	29.2909660000
O68	10.9137350000	50.0421020000	28.9245740000
H69	17.0271050000	49.4809750000	24.2815000000
H70	17.2167880000	48.2062120000	23.0761920000
H71	16.4095230000	47.8528050000	24.6173720000
H72	16.6744500000	48.0837030000	29.0578080000
H73	16.0654050000	47.0987920000	30.3742540000
H74	16.5078290000	48.9857830000	32.0004680000
H75	17.1336360000	49.9731490000	30.6722780000
H76	15.2479940000	49.6423140000	27.9226280000
H77	11.5921200000	48.6300130000	31.1541190000
H78	13.9068100000	47.8242000000	31.5687950000
H79	5.7908440000	45.4576150000	26.4431700000
H80	4.5808890000	46.4408910000	27.2231990000
H81	7.6723500000	44.2652840000	23.0228140000
H82	8.8799590000	43.9047610000	21.8066250000
H83	4.7960680000	46.0603920000	24.1626150000
H84	3.6792200000	45.1895230000	25.2297790000
H85	10.2805620000	42.7539380000	23.6320730000
H86	8.9066260000	42.9887210000	24.7331210000
H87	3.9158580000	48.8248460000	27.5023020000
H88	4.0438850000	49.9760450000	26.1771440000
H89	4.8010200000	50.3414110000	27.7426230000
H90	12.0353610000	45.5559110000	20.9795430000
H91	10.8660910000	44.2429030000	21.1617850000
H92	12.3160400000	44.2808370000	22.1699060000
H93	9.6066770000	54.3150930000	20.1673410000
H94	9.4401240000	52.5927320000	19.8856630000

H95	11.7607070000	53.7202090000	21.2795630000
H96	14.1980850000	52.6162510000	22.7881540000
H97	9.9862100000	53.0863670000	30.6711570000
H98	10.1274250000	54.6257620000	29.7834570000
H99	11.3816990000	54.1473670000	30.9128420000
H100	7.8444800000	53.2308620000	29.2963490000
H101	7.4004590000	53.4823860000	27.6007950000
H102	8.6970770000	54.4328040000	28.3135590000
H103	6.7712070000	51.4500750000	27.1281590000
H104	7.2633150000	45.5295760000	24.7677980000
H105	6.9767600000	40.6252160000	23.0833550000
H106	2.4711020000	46.6416980000	26.6231860000
H107	13.9160060000	52.5242090000	20.4784290000
H108	13.2001410000	49.7495800000	21.5667740000
H109	14.0291490000	50.1089140000	20.0463220000
H110	9.6964080000	53.9668110000	22.6352370000
H111	8.1700320000	53.5509630000	21.8783280000
H112	10.4929640000	52.0103260000	24.4934390000
H113	8.0262390000	50.7591220000	21.3340750000
H114	8.4000370000	48.7588000000	22.8522020000
H115	13.9846750000	54.5007640000	25.5256690000
H116	12.6201080000	54.3331010000	24.4111490000
H117	13.1252940000	52.9592440000	25.4055530000
H118	11.8744380000	55.5835190000	26.4509970000
H119	11.0569840000	54.0201980000	26.4076430000
H120	11.8925810000	51.4373070000	19.5173100000
H121	11.2832150000	51.1454690000	21.1516900000
H122	15.1220490000	51.7768040000	26.4528560000
H123	15.8173350000	51.5414070000	24.8372320000
H124	16.1976790000	50.4090140000	26.1405490000
H125	13.0605110000	47.2692040000	22.6789600000
H126	12.1154790000	52.3125790000	29.5694640000
H127	14.8690660000	48.0877640000	22.6219800000
H128	12.9850740000	51.7054290000	27.2554430000
H129	12.7925360000	51.8574640000	23.0899340000
H130	17.9754160000	48.4835730000	31.1428250000
N131	10.0392800000	46.5841980000	27.0972140000
O132	10.4315720000	47.7586610000	27.0734920000
N133	10.3070320000	45.9676330000	28.5491800000
O134	10.4561870000	46.8119300000	29.3632910000
